# Amyloid precursor protein and presenilin‐1 knock‐in immunodeficient mice exhibit intraneuronal Aβ pathology, microgliosis, and extensive neuronal loss

**DOI:** 10.1002/alz.70084

**Published:** 2025-04-07

**Authors:** Pravin Yeapuri, Jatin Machhi, Emma G. Foster, Rana Kadry, Shaurav Bhattarai, Yaman Lu, Susmita Sil, Roshan Sapkota, Shefali Srivastava, Mohit Kumar, Tsuneya Ikezu, Larisa Y. Poluektova, Howard E. Gendelman, Rodney Lee Mosley

**Affiliations:** ^1^ Department of Pharmacology and Experimental Neuroscience Center for Neurodegenerative Disorders College of Medicine University of Nebraska Medical Center Omaha Nebraska USA; ^2^ Department of Neuroscience Mayo Clinic Florida Jacksonville Florida USA

**Keywords:** Alzheimer's disease, APP, human immune reconstitution, immunodeficient NOG background, intraneuronal Aβ deposition, knock‐in mice, microgliosis, neuronal loss, PS1, translational AD research models

## Abstract

**INTRODUCTION:**

Transgenic mice overexpressing familial Alzheimer's disease (AD) mutations (FAD) show non‐physiological traits, and their immunocompetent backgrounds limit their use in AD immunotherapy research. Preclinical models that reflect human immune responses in AD are needed.

**METHODS:**

Using CRISPR‐Cas9, we developed single (NA) and double (NAPS) knock‐in (KI) amyloid precursor protein (APP)^KM670,671NL^ (Swedish) and presenilin 1 (PS 1)^M146V^FAD mutations on an immunodeficient NOG (NOD.Cg‐Prkdc^scid^Il2rg^tm1Sug^/JicTac) background. The models were confirmed by Sanger sequencing and evaluated for AD‐like pathology.

**RESULTS:**

Both NA and NAPS mice developed pathology without overexpression artifacts. Mutation‐induced upregulation of APP‐CTF‐β led to intraneuronal human amyloid beta (Aβ) (6E10) deposits and amyloid‐associated microgliosis as early as 3 months, which increased with age. The addition of the PS 1^M146V^ mutation doubled the amyloid load. The models displayed broad neuronal loss, resulting in brain atrophy in older mice.

**DISCUSSION:**

These models replicate intraneuronal amyloid pathology and, with human immune reconstitution potential, enable novel studies of human immune responses in AD.

**Highlights:**

- A novel Alzheimer's disease (AD) knock‐in (KI) mouse was developed and characterized on an immunodeficient NOG background. The model provides a platform for human immune studies and the evaluation of immunotherapies for AD.
- The KI mice demonstrate intraneuronal Aβ deposits and amyloid‐associated microglial reactions.
- KI mice demonstrate extensive neuronal loss.
- Human immune reconstitution enables studies of infectious AD co‐morbidities, such as the human immunodeficiency and herpes simplex viruses.

**Abbreviations**
22C11: human/mouse reactive anti‐Aβ antibody
3xTg: transgenic for Swedish APP, P301L Tau, and M146V KI mutation of PS1
5xFAD: transgenic for Swedish, Florida and London APP, and M146L and L286L PS1
6E10: human specific anti‐Aβ antibody
AD: Alzheimer's disease
APP: human amyloid precursor protein
App: mouse amyloid precursor protein
APP/PS1KI: transgenic for Swedish, London APP and KI of two PS1 mutations
APP^KM670,671NL^
: Swedish mutation of APP
APP^NL^
: KI of Swedish APP mutation
APP^NL‐F^
: KI of Swedish and Iberian APP mutations
APP^NL‐G‐F^
: KI of Swedish, Arctic, and Iberian APP mutations
Aβ: amyloid beta
CD34‐NOG‐hIL‐34: NOG mice with IL‐34 transgene that develop human‐like microglia
CRISPR: clustered regularly interspaced short palindromic repeats
CTF‐α: c‐terminal fragment α
CTF‐β: c‐terminal fragment β
FAD: familial Alzheimer's disease
FL‐APP: full length‐APP
hAβ‐KI: mouse Aβ substituted with wild‐type human isoform
HSC: hematopoietic stem cells
Iba1: ionized calcium binding adaptor molecule 1
KI: knock‐in
MAP2: microtubule‐associated protein 2
NA: single knock‐in APP^KM670,671NL^
NAPS: double knock‐in APP ^KM670,671NL^ X PS1^M146V^ mice
NOG: immunodeficient NOD/Shi‐scid/IL‐2Rγnull mice
PD: Parkinson's disease
PS1: human presenillin 1
Ps1: mouse presenillin 1
PS1^M146V^
: PS1 mutation
Teff: effector T cell
Treg: regulatory T cell

## 1 BACKGROUND

Alzheimer's disease (AD) is a progressive neurodegenerative disorder and the leading cause of dementia worldwide. It is characterized by the accumulation of amyloid beta (Aβ) plaques, tau neurofibrillary tangles, synaptic dysfunction, neuroinflammation, and neuronal loss.^1^ Despite significant advancements in understanding AD mechanisms, translating preclinical findings into effective human therapies remains challenging.^1^ Animal models for translational AD research must closely reflect human disease biology.^2^, ^3^

More than 170 AD mouse models exist, most of which are transgenic mice overexpressing human familial AD mutations (FAD) associated with AD neurodegeneration driven by non‐endogenous promoters.^4^, ^5^, ^6^ In addition to overexpression‐based artifacts, the transgene insertion sites in these mice have not been accurately mapped, complicating the clinical relevance of the observed phenotypes for translational research. Furthermore, the complex interplay between the human brain, host genetics, and immunity complicates cross‐species translation of disease mechanisms and further creates challenges in modeling cause‐and‐effect disease processes.^7^ This discrepancy has led to successful therapeutic results in AD models not accurately recapitulated in human clinical trials.^6^ One notable unforeseen challenge in AD translational research is the development of meningoencephalitis in humans associated with anti‐Aβ antibody immunotherapies.^8^, ^9^ While preclinical studies in transgenic mice have shown effective plaque removal and cognitive improvement, the brain infiltrating effector T cell (Teff)‐induced meningoencephalitis was not accurately recapitulated in mice.^8^, ^9^

The available transgenic AD models that incorporate the supra‐physiological expression of disease‐linked autosomal‐dominant mutations have significant limitations in translational research.^1^, ^2^, ^10^ Therefore, animal models that accurately recapitulate the underlying molecular and neuroinflammatory pathways associated with AD are needed. Species‐specific differences in innate and adaptive immunity affect plaque clearance and systemic neurobiology, introducing significant variables in developing preclinical AD models.^11^, ^12^ Suitable models must incorporate human‐like neuroimmune crosstalk, particularly involving adaptive immune responses in AD pathology.^13^, ^14^, ^15^ Knock‐in (KI) rodent models have emerged as a promising alternative to address this gap, where familial AD mutations are knocked into rodent homologs, and endogenous promoters drive pathological protein expression.^16^, ^17^, ^18^ However, when expressed in immunocompetent backgrounds, human AD protein engagements with a rodent immune system affect the clinical relevance of immunotherapies. Those therapies tested in these mice represent a knowledge gap in current AD immunotherapy research.^19^ Recent studies highlighted a key role of adaptive immunity in AD pathogenesis.^20^, ^21^ Disease‐exacerbating^22^ and neuroprotective roles of amyloid‐specific regulatory T cells (Treg)^23^ were shown in human amyloid precursor protein (APP)/human presenillin 1 (PS1) transgenic mice. Determining translation of these findings is limited and underscores the urgent need for relevant humanized animal models for study. Brain organoids aimed to model AD pathologies are limited by their absence of vasculature, presence of immature cells, and challenges in accurately replicating age‐associated disease.^24^, ^25^ These limitations further emphasize the need for next‐generation murine models that bridge immunological gaps in AD research.

To address these challenges, founder lines carrying amyloid precursor protein (APP)^KM670,671NL^ and presenilin 1 (PS 1)^M146V^ mutations were developed on an immunodeficient Cg‐Prkdc^scid^ Il2rg^tm1Sug^/JicTac (NOG) mice background, using clusetered regularly interspaced short palindromic repeats (CRISPR)‐CRISPR‐associated protein 9 (Cas9) KI technologies. These single‐mutant NOG/APP (NA) and double‐mutant NOG/APP/PS1 (NAPS) KI mice exhibited clinically relevant AD pathologies, including intraneuronal amyloid deposits, microgliosis, and broad neuronal loss. Importantly, these models overcome previous limitations in generating human disease‐relevant insights and testing immunotherapeutic interventions by allowing the reconstitution of a human immune system. Notably, by allowing the examination of the human adaptive immune system's interactions with human amyloid pathology, these models provide valuable insights into immune‐associated AD comorbidities and serve as tools for evaluating AD immunotherapies.

## 2 METHODS

### 2.1 Study approvals

Animal procedures strictly followed the Institutional Animal Care and Use Committee (IACUC) guidelines, which approved protocols at the University of Nebraska Medical Center (UNMC), and the Institutional Guidelines (11004), which were approved by the Animal Experimentation Committee of the Central Institute of Experimental Animals (CIEA).

### 2.2 Animals

Genetic manipulations were done on non‐obese diabetic (NOD) mice and then backcrossed to create NOD.Cg‐Prkdc^scid^Il2rg^tm1Sug^/JicTac mice (NOG) mice. These were purchased from Taconic Biosciences (Germantown, NY) and used to generate the KIs. Mice were housed in micro isolator cages with corncob bedding and crinkle paper for enrichment in the laboratory animal facility at UNMC and maintained on a 12‐h light‐dark cycle. Sterile water and rodent feed (2019, Envigo Teklad diet, Madison, WI) were provided ad libitum. Cages were changed weekly, and animals were checked daily for health concerns.

**RESEARCH IN CONTEXT**

1. **Systematic review**: The authors reviewed the literature on Alzheimer's disease (AD) relevant to preclinical models. Transgenic animal models rely on the overexpression of AD proteins and have limited translational value. While knock‐in (KI) models show promise, their immunocompetent backgrounds limit studies of human immunotherapies. No models exist with a functional human immune system, which includes organoids. Relevant citations are provided.
2. **Interpretation**: We developed KI mouse models on an immunodeficient NOG background, allowing human immune system reconstitution. The models show AD pathology without overexpression artifacts. Intraneuronal amyloid and microgliosis are present. The created models exhibit extensive neuronal loss. The current work provides a novel system for studying AD pathology in the context of adaptive human immunity.
3. **Future directions**: This study uses immunodeficient KI models to explore the role of the human immune system in AD pathobiology. It will also explore disease‐specific immune therapies and facilitate studies of the molecular mechanisms underlying a range of disease‐combating therapies.

### 2.3 KI mice development

KI mice harboring APP^KM670,671NL^ or PS 1^M146V^ mutations and partially humanized APP and PS1 sequences were generated using CRISPR‐Cas9 technology. RNA guides were selected using the CRISPR Web tool for off‐ and on‐target scoring. Guide RNAs were designed to precisely excise mouse wild‐type genome sequences using Cas9 protein and DNA repair machinery, allowing successful donor DNA replacement. NOD mice were mated, and fertilized eggs were harvested from pregnant females. Humanized mouse genomic sequences of AD pathological mutations were then microinjected into these fertilized eggs. After microinjection, eggs were transplanted back into pseudo‐pregnant recipient mice. The offspring were genotyped by Sanger sequencing to identify the presence of the gene of interest. Heterozygous offspring were then mated with NOG mice to generate homozygous single KI APP^KM670,671NL^ and single KI PS 1^M146V^ mice on an NOG background. Further, double KI APP^KM670,671NL^ PS 1^M146V^ mice were generated by crossing single KI mice and breeding them to homozygosity for both genes. This report refers to these as NA and NAPS mice, respectively.

### 2.4 Genotyping

Sanger sequencing confirmed successful KI of human APP and PS1 familial AD mutations. Ear tissue was collected at weaning and digested using the alkaline lysis buffer 0.4 M NaOH and 0.2 mM ethylenediaminetetraacetic acid (EDTA). Samples were heated at 95°C for 1 h until fully digested. Samples were then neutralized with a neutralization buffer (40 mM Tris‐HCl) and centrifuged at 10,000 x *g* for 5 min. Supernatants were used for DNA amplification. Polymerase chain reaction (PCR) was performed to amplify the gene of interest (APP or PS1) using GoTaq Hot Start Green Master Mix (cat. M5123, Promega) and forward and reverse primers specific to each gene (APP for GGCGGTCACACTAACGGATG, APP rev: GACACAGGACAAGCCACGAG, PS1 for: ACTCATTAAACCGCAGACCTCAC, PS1 rev: CACACACAAGGACAACCCATAGG). PCR products were confirmed by gel electrophoresis on a 1% agarose gel, then purified using the ZR DNA Sequencing Clean‐up Kit (cat. D4051, Zymo Research). Following purification, samples were mixed with the forward primer for the gene of interest and submitted to Azenta Life Sciences (Burlington, MA) for sequencing. SnapGene Viewer was used to visualize nucleotide sequences and identify mutations to determine whether mice were heterozygous or homozygous for human familial mutations, APP, PS1, or both.

### 2.5 Tissue collection

Mice were humanely euthanized at 3, 6, or 9 months of age, and brain tissues were collected for further analysis. Under terminal anesthesia (Fatal Plus, pentobarbital), mice were perfused via cardiac puncture with phosphate‐buffered saline (PBS). Following perfusion, the brain was carefully removed. The right hemisphere was separated and placed in 4% paraformaldehyde (Sigma‐Aldrich) in PBS. The left hemisphere was dissected, and the cortex and hippocampus were frozen and stored at −80°C for biochemical analyses.

### 2.6 Western blot analyses

Frozen brain tissues were analyzed for expression of APP and PS1 proteins using Western blot analysis.^26^ Tissues were homogenized in 50 mM Tris‐HCl (pH 8.0) containing 150 mM NaCl, 50 mM EDTA, 1% Triton X‐100, and protease and phosphatase inhibitor cocktail (cat. 78442, Thermo Fisher Scientific). Lysates were centrifuged at 20,000 × *g*  for 1 h at 4°C, and supernatants were collected. Protein concentrations were determined with the Pierce BCA Protein Assay Kit (cat. 23227, Thermo Fisher Scientific). Ten to fifteen micrograms of protein were run on a 10% or 15% sodium dodecyl sulfate (SDS)‐polyacrylamide gel, and separated proteins were transferred to an activated 0.2 µM polyvinylidene fluoride (PVDF) membrane. Following the transfer, membranes were blocked in 5% non‐fat milk for at least 1 h, then incubated in individual primary antibodies on a shaker at 4°C overnight. Primary antibodies included anti‐β‐amyloid, 1‐16 (1:1000, clone 6E10, cat. 83001, BioLegend), anti‐APP (1:1000; clone 22C11, cat. MAB348, EMD Millipore), anti‐APP C‐terminal (1:1000; cat. A8717, Millipore Sigma‐Aldrich), anti‐presenilin‐1 (1:1000; cat. NBP1‐33611, Novus Biologicals), anti‐PS1 C‐terminal (1:1000; cat. MAB5232, Millipore Sigma‐Aldrich), and anti‐β‐actin (1:2000; cat. sc‐47778, Santa Cruz Biotechnology). After incubating overnight, the primary antibody was removed, and membranes were washed with Tris‐buffered saline with 0.1% Tween 20 detergent (TBST) three times (10 min each wash). Membranes were then incubated in secondary antibodies at room temperature for 2 h. Secondary antibodies included goat anti‐rabbit immunoglobulin G (IgG) H&L (horseradish peroxidase [HRP]) (1:10,000; cat. ab205718, Abcam) and goat anti‐mouse IgG H&L (HRP) (1:10,000; cat. ab205719, Abcam). Following incubation with secondary antibodies, membranes were washed with TBST three times (10 min each), then developed with SuperSignal West Pico PLUS Chemiluminescent Substrate or SuperSignal West Femto Maximum Sensitivity Substrate (Thermo Fisher Scientific) and imaged on an iBright 750 Imaging System. Analysis of band intensity was performed using ImageJ software.

### 2.7 Aβ enzyme‐linked immunosorbent assay (ELISA)

Snap‐frozen mouse hippocampus was homogenized in 50 mM Tris‐HCl (pH 7.6) containing 150 mM NaCl and a protease inhibitor. Lysates were centrifuged at 20,000 × *g*  for 60 min at 4°C, and the supernatant was collected to detect a soluble fraction of Aβ_42_. For detecting insoluble fractions of Aβ_42_, pellets were dissolved using 6 M  guanidine‐HCl and centrifuged at room temperature at the same speed and time. According to the manufacturer's instructions, Aβ_1‐42_ loads in the brain cortex were quantified using an ELISA kit (Quantikine ELISA catalog No. DAB142, R&D systems).

### 2.8 Immunohistochemical (IHC) and immunofluorescence (IF) staining

After transcardial perfusion, the right hemisphere of mouse brains was immersed in freshly depolymerized 4% paraformaldehyde in PBS for 24 h at 4°C. Brains were then transferred to 70% ethanol until ready for processing. Brains were processed overnight in an Epredia STP 120 Spin tissue processor (Thermo Fisher Scientific) and embedded in paraffin blocks. Five micrometer‐thick brain sections were collected directly onto slides for IHC and IF staining. IHC slides were deparaffinized, and antigen retrieval was performed using the Trizol reagent as previously described using a heat‐induced method.^27^ Brain sections were blocked with 10% normal goat serum following antigen retrieval to prevent non‐specific staining. Sections were then incubated with primary mouse monoclonal antibodies against human Aβ (6E10 antibody; cat. 803001, BioLegend) or anti‐amyloid fibrils OC antibody (cat. AB2286, Sigma‐Aldrich) overnight at 4°C. Following washing, sections were incubated with HRP‐conjugated anti‐mouse secondary antibody (cat. GTX83398, GeneTex) for 1 h at room temperature. 3,3′‐Diaminobenzidine (DAB) was used as a detection chromogen (cat. D4293, Millipore Sigma), and hematoxylin (cat. 10015‐074, VWR) was used for nuclear counterstaining. Slides were imaged using a Zeiss Axioscan 7 whole slide imaging system. For thioflavin staining, tissue slides were incubated in 50 µM of Thioflavin T diluted (cat. 596200, Sigma‐Aldrich) in PBS for 20 min. The slides were washed twice with PBS for 5 min, and the coverslipped.^28^

For IF staining blocked brain sections were incubated overnight at 4°C with rabbit polyclonal antibody against Iba1 (cat. 01327691, Fujifilm), a guinea pig monoclonal antibody against NeuN (cat. 266004, Synaptic Systems), or mouse monoclonal antibody against MAP‐2 (cat. 13‐1500, Invitrogen). Following washing, tissue was incubated with Alexa 488‐conjugated goat anti‐rabbit IgG (cat. A11008, Invitrogen), Alexa 568‐conjugated goat anti‐guinea‐pig IgG (cat. A11075, Invitrogen), or Alexa 488 conjugated goat anti‐mouse IgG (cat. 32723, Invitrogen) secondary antibody for 1 h at room temperature. DAPI was used as nuclear staining (cat. D1306, Thermo Fisher Scientific). Images were taken using a Zeiss Axioscan 7 whole slide imaging system. Images were analyzed using HALO AI software (v3 version). The Indica Labs—Area Quantification v2.4.3 module was used for Aβ staining area quantification. The percentage of 6E10 staining area output was used for analysis. Similarly, for NeuN and MAP‐2 staining area quantification, the Indica Labs—Area Quantification FL v2.3.4 module was used. The percentage of NeuN or MAP‐2 staining area output was used for the analysis. The Indica Labs—Microglial Activation FL v1.0.6 module was used for microglial activation analysis. Microglial cells with less than 15 processes and a total process area of more than 15 µm^2^ were scored as activated to identify activated microglia phenotype.

### 2.9 RNA sequencing and bioinformatics

Flash‐frozen cerebral cortex tissue from mice at 3, 6, and 9 months (3 mice/group) was used for transcriptomic analysis. Frozen tissues were thawed on ice and homogenized using a TissueLyser II (Qiagen) and 5 mm stainless steel beads (cat. 69989, Qiagen). Total RNA was extracted using the RNeasy Mini Kit (cat. 74104, Qiagen). RNA quality was assessed with an Agilent 2100 Bioanalyzer using the RNA 6000 Nano Kit (cat. 5067‐1511, Agilent). All RNA samples were sequenced on the Illumina HiSeq 2000 platform using the Novaseq 6000 SP‐200 cycle (paired‐end, 101 bp reads) (cat. 0028315, Illumina). The RNA sequencing analysis was performed using the nf‐core v3.12.0 pipeline in Nextflow v23.04.1.^29^, ^30^ The nf‐core pipeline trims the fastq files using cutadapt and trimgalore to remove adapters, terminal unknown bases (Ns), and low‐quality 3′ regions (Phred score < 30).^31^ The trimmed fastq files were processed by FastQC, a quality control tool for high‐throughput sequence data, available online at http://www.bioinformatics.babraham.ac.uk/projects/fastqc/ for quality control.^32^ The trimmed fastq files were processed by newly developed standard pipelines utilizing STAR,^33^ and the aligner and RSEM^34^ were tools used for annotation and quantification at the gene level. The trimmed fastq files were mapped to the mm10/GRCm38 mouse reference genome. The raw counts were used for differentially expressed gene (DEG) analysis by DESeq2.^35^ The Benjamini–Hochberg (BH) adjusted *p*‐values were provided to adjust for multiple‐testing caused false discovery rate (FDR). We used iDep 2.0 software for principal component pathway (PCA) pathway analysis, k‐clustering, and pathways/mechanisms contributing to the disease progression; we used the Ingenuity Pathway Analysis (IPA, Qiagen). *P* value ≤ 0.05 and the absolute fold change of ≥ 1.5 were used to select the significantly altered pathways.

### 2.10 Behavioral testing

Novel object recognition tests and a Y‐maze were used to assess KI mice's memory function. Approximately 8‐ and 12‐month‐old NA and NAPS mice and age‐matched NOG controls were used for behavioral testing. A novel object recognition test was used to evaluate visual recognition memory.^15^ In the novel object recognition test, mice were acclimated to a square arena with no objects present for 10 min. Following a 90‐min break, mice were given three 5‐min object acclimation trials with two identical objects (familiar objects). These were done with 90‐min inter‐trial intervals. Ten minutes after the last object acclimation trial, mice underwent a 5‐min novel object test. In the test, one of the familiar objects was replaced by a novel object to test visual recognition memory. ANY‐maze software was used to track mice in the arena and determine the time spent exploring each object. The discrimination index, calculated as (time spent with the novel object / total object exploration time) × 100, was recorded for all animals.

The Y‐maze was used to evaluate spatial learning and memory using the short‐term alternations method.^36^ The maze consisted of three 39.5 × 8.5 × 13 cm arms with a 120° angle between arms (arms A, B, and C). Mice were placed at the end of one arm facing the wall and allowed to freely explore the maze for 8 min. The number and order of arm entries were recorded using ANY‐maze tracking software. An arm entry was considered when all four mouse paws entered the arm. Spontaneous alternations were defined as consecutive entries into all three arms (i.e., ABC, BCA, or CAB, but not BAB). The percentage of spontaneous alternations was calculated as [number of spontaneous alternations/(total number of entries − 2)] x 100. Mice were given two 8‐min trials, one each day for 2 days. The first day was considered an acclimation trial, and the second was considered a test trial. Percent spontaneous alternations were calculated only for the test trial.

### 2.11 Magnetic resonance imaging (MRI)

Fifteen mice were scanned for MRI (4 NOG, 8 NA, and 3 NAPS mice) at 9 months of age. MRI was conducted on a Bruker 7 T scanner (PharmaScan). Mice were anesthetized using isoflurane carried with oxygen at a 1 L/min flow rate. Breathing was monitored during scanning. High‐resolution T2‐weighted images were acquired using TurboRARE with repetition time/echo time (TR/TE) = 3000/36 ms, RARE factor = 4, number of slices = 25, slice thickness = 0.5 mm, field of view (FOV) = 20 × 20 mm^2^, and matrix = 256 × 256. Ventricle volumes were measured using region‐of‐interest (ROI) analysis in ImageJ.

### 2.12 Human immune reconstitution

Newborn pups (post‐natal day 0–3) were irradiated with 1 Gy (RS 2000 x‐ray Irradiator, Rad Source Technologies, Inc., Suwanee, GA, USA). After 4 h of irradiation, pups were intrahepatically injected with 10^5^ CD34+ HSC (hematopoietic stem cells). For comparison, NOG mice were also reconstituted with CD34+ HSPCs. Engraftment of human leukocytes was examined by flow cytometry analysis of blood samples collected from cheek vein bleeds 12 weeks post engraftment.

### 2.13 Flow cytometry

Blood samples were collected from a facial vein in EDTA‐containing tubes (BD Microtainer, Franklin Lakes, NJ, USA) and centrifuged at 1800 rpm for 8 min. Blood cells were reconstituted in a fluorescence‐activated cell sorting (FACS) buffer (2% FBS in phosphate‐buffered saline) and incubated for 30 min at 4°C with a cocktail of antibodies against human immune cell markers (fluorophore), CD45+ (fluorescein isothiocyanate [FITC]), CD3+ (Alexa Fluor 700 [AF700]), CD19+ (phycoerythrin‐cyanin 5 [PE‐Cy5]), CD4+ (allophycocyanin [APC]), CD8+ (Brilliant Violet 421 [BV421]), and CD14+ (phycoerythrin [PE]). All antibodies and isotype controls were obtained from BD Biosciences, USA. Red blood cells were lysed by FACS lysing solution (BD Biosciences, USA). Stained cells were washed with FACS buffer and fixed with 2% paraformaldehyde. Data acquisition was carried out with acquisition software FACS Diva v6 (BD Biosciences, USA) in a BD LSR2 flow cytometer, and data were analyzed using FLOWJO analysis software v10.2 (Tree Star, USA). Gates were assigned according to the appropriate control population.

## 3 RESULTS

### 3.1 Development of a single KI NA mouse

**FIGURE 1:**
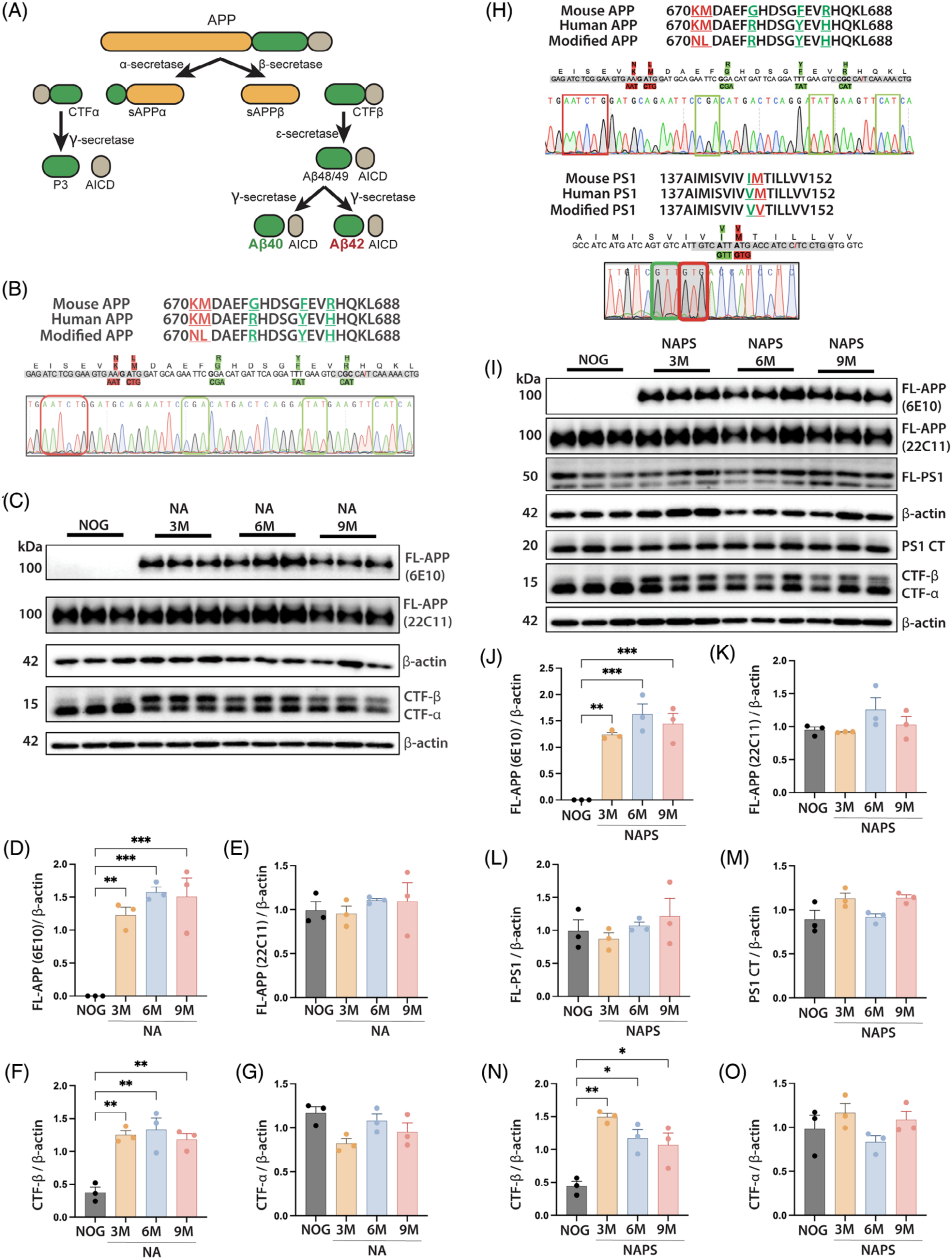
Development and characterization of single knock‐in (KI) APP^KM670,671NL^ (NA) and double KI APP^KM670,671NL^ x PS 1^M146V)^(NAPS) mice. (A) Schematic representation of pathological APP processing. (B) Details of nucleotide changes and corresponding amino acids in APP of NA mice are summarized. Sanger sequencing confirms the successful replacement of pathological mutation (red box) and humanized (green box) nucleotides in mouse APP, exon 16. (C) Cortical tissues from immunodeficient NOD/Shi‐scid/IL‐2Rγ^null^ (NOG) or NA mice were obtained at 3, 6, and 9 months of age and assessed by Western blot for human (6E10) or human/mouse (22C11) full‐length (FL)‐APP, APP‐CTFβ, and APP‐CTFα fragment quantitation from Western blots of (D) human and (E) mouse FL‐APP, (F) CTFβ, and (G) CTFα fragments. Quantitation was normalized to β‐actin expression. (H) Details of nucleotide changes and corresponding amino acids in APP and PS1 mice are summarized. Sanger sequencing confirms the successful replacement of pathological mutation (red box) and humanized (green box) nucleotides in mouse APP, exon 16 and PS1, exon 5. (I) Cortical tissues from NOG or NAPS mice were obtained at 3, 6, and 9 months of age, and Western blots were used for APP and PS1 fragments in NAPS mice compared with NOG controls. Differential expression of APP and PS1 fragments in NOG and NAPS mice were quantitated for (J) human FL‐APP (6E10), (K) human/mouse FL‐APP (22C11), (L) FL‐PS1, (M) PS1‐CT, (N) CTFβ, and (O) CTFα fragments. Quantitation was normalized to β‐actin expression. Data are represented as mean ± SEM, *N* = 3 mice per group by one‐way ANOVA and Tukey's post hoc tests for *p < 0.05, **p < 0.01, and ***p < 0.001.

The genomic DNA sequences of the mouse and human *APP* were searched in the NCBI database, and the sequence for the Aβ_42_ peptide encoded by the sequence of exon 16 of the *APP* gene was analyzed and compared. To develop an *APP* KI mice containing the Swedish double mutation of familial AD, the two mutations were knocked into exon 16 (K670N substitution and M671L substitution).^37^ Furthermore, we noticed additional differences between the mouse and human *APP*, exon 16. To ultimately humanize exon 16, mutations substituting G676R, F681Y, and R684H were knocked in. The differential APP cleavage that leads to the development of amyloid plaque is shown in Figure 1A. Figure 1B provides a schema indicating the amino acid and nucleotides changed on exon 16 to introduce the Swedish mutation (highlighted red) and humanize (highlighted green) the exon 16 on the mouse *App* gene. Sanger sequencing confirmed the successful KI of the Swedish *APP* mutation and humanization of Exon 16 on NOG background mice (Figure 1B). The KI NA mice were maintained as homozygous lines. In the first‐generation transgenic *APP* mouse models, familial AD mutations are randomly inserted into the mouse genome, and non‐endogenous promoters drive gene overexpression.^18^ In our mice, the familial AD genes were knocked into the same gene locus on the mouse *APP* homologs. Therefore, gene expression is driven by endogenous promoters, and overexpression‐based artifacts are avoided. Under physiological conditions, full‐length APP is cleaved by different secretase enzymes (α, β, γ, and ɛ) to produce APP fragments (APP‐NTF n‐terminal fragment), ‐CTF (c‐terminia fragment), and AICD (APP intracellular domain) and Aβ_40_ (Figure 1A). However, in AD, differential cleavage of the APP fragments leads to the development of toxic Aβ_42_ production, which subsequently aggregates into neurotoxic Aβ plaques (Figure 1A).^38^ Aβ_42_ is selectively overproduced in AD patients, while wild‐type *APP* production is unaffected.^39^ To characterize the overexpression‐free features of our model, we first confirmed the expression of chimeric *APP* using an antibody that recognizes explicitly human Aβ (1‐16) (clone 6E10). Western blot analysis of cortical tissue from mice 3, 6, and 9 months of age confirmed the stable expression of chimeric full‐length *APP* (FL‐APP), while no expression was observed in NOG mice (Figure 1C,D,E). An antibody that recognizes both mouse and human Aβ (1‐16) (clone 22C11) was used to evaluate the lack of overexpression‐based artifacts and physiological levels of *APP* gene expression. Western blot analysis of cortical tissues from mice 3, 6, and 9 months of age showed similar levels of FL‐APP (22C11) in both NA and NOG mice, thus confirming the lack of overexpression and physiological levels of *APP* gene expression in the NA mice.

Further, the Swedish mutation of *APP* affects the proteolytic sites of the β‐secretase. It increases the production of pathological Aβ by inducing differential cleavage of APP fragments (CTF‐α and CTF‐β) (Figure 1A). The mutation‐induced differential cleavage of APP fragments was confirmed by significantly increased levels of APP‐CTF‐β fragments in NA mice (Figure 1A,F). At the same time, APP‐CTF‐α was comparable to NOG mice (Figure 1A,G).

### 3.2 Development of double KI NAPS mice

The combination of *APP* with other mutations, such as *PS1* familial AD, has been routinely used in the development of transgenic mice. This was done to enhance the expression of pathological AD.^18^ Point mutations in the *PS1* gene are a significant cause of familial AD that results in a selective increase in the production of amyloid pathology.^40^ The *PS1* familial AD mutation (M146 V) is positioned on exon 5 of the *PS1* gene. Like KI NA mice (Figure 1B), an M146 V KI substitution generated a chimeric *PS1* gene carrying the familial human AD *PS1* mutation on exon 5.

Furthermore, to humanize exon 5, we knocked in I145 V substitutions. Figure 1H provides a schema indicating the amino acid and nucleotides changed on exon 6 to KI the familial human *PS1* mutation (highlighted red) and humanize the sequence (highlighted green). Sanger sequencing confirmed the successful KI of the *PS1* mutation and the humanization of exon 6 on the NOG background. Homozygous KI PS 1^M146V^ (NPS) mice were crossed with homozygous NA mice to develop double NAPS KI mice on the NOG background. Sanger sequencing confirmed the presence of homozygous APP^KM670,671NL^ and PS 1^M146V^ KI and humanization of the exon containing the mutations (Figure 1H). Like NA mice, the double KI NAPS mice were evaluated for physiological levels of chimeric *APP* and *PS1* gene expression and differential APP cleavage. Western blot analysis of cortical tissues from NAPS mice at 3, 6, and 9 months of age demonstrated the stable expression of chimeric FL‐APP (human‐specific 6E10 mAb) compared to control NOG mice (Figure 1I,J).

**FIGURE 2:**
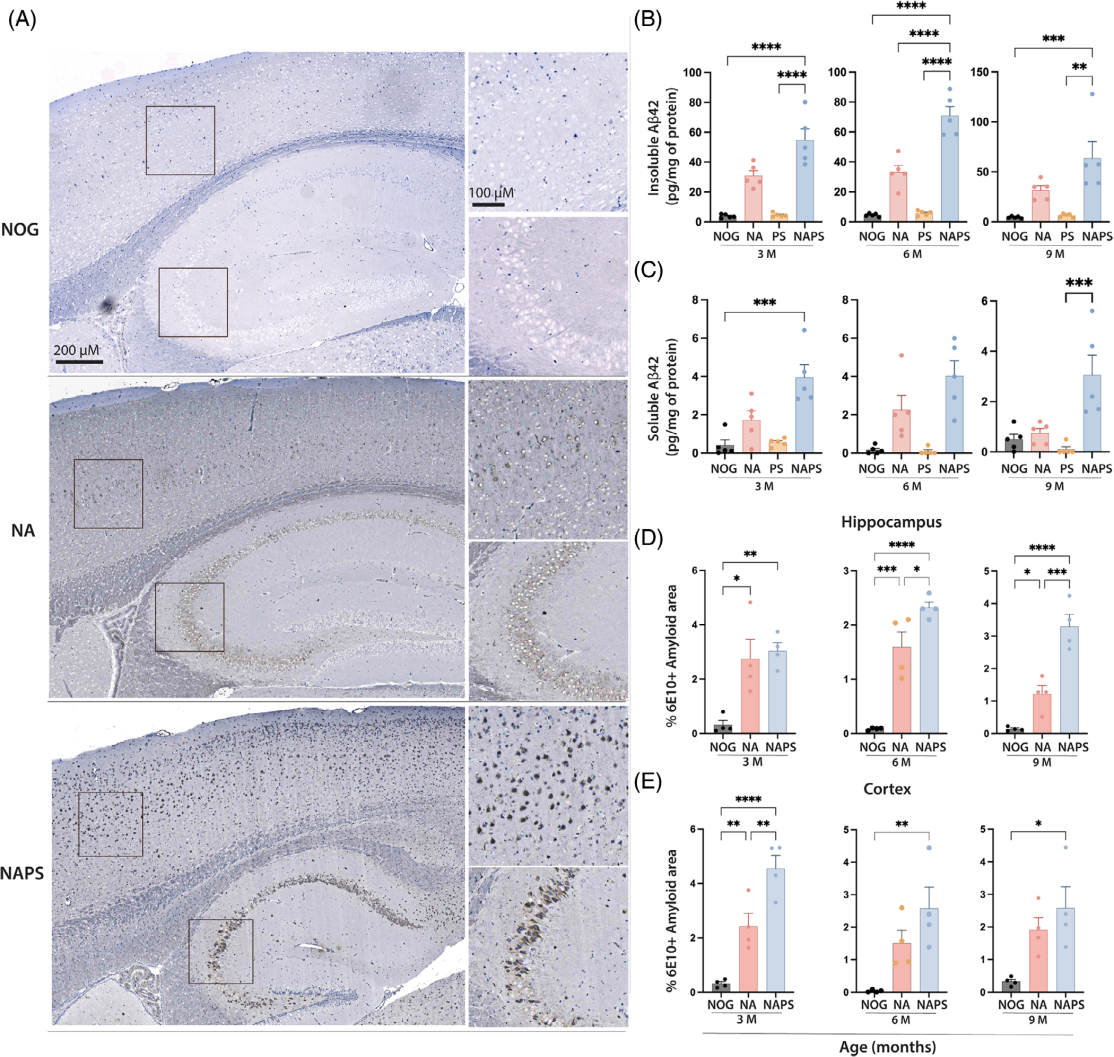
Characterization of amyloid deposition in single knock‐in (KI) APP^KM670,671NL^ (NA) and double KI APP^KM670,671NL^ x PS 1^M146V^ (NAPS) mice. Brains were harvested from 3‐, 6‐, and 9‐month‐old immunodeficient NOD/Shi‐scid/IL‐2Rγ^null^ (NOG), NA, and NAPS mice. (A) Representative immunohistochemical (IHC) images (5 µm sagittal sections) of human‐Aβ deposition (6E10 antibody) in 6‐month‐old mice (scale bar = 200 µm) using heat‐induced epitope retrieval. Areas of human‐Aβ deposition in the cortex and hippocampus are highlighted by inserts (scale bar = 100 µM). Quantitation of human‐Aβ deposition by (B) human Aβ_42_ ELISA using soluble (Tris‐HCL) and (C) insoluble (Gu‐HCl) protein fractions of brain cortical tissue. IHC quantitation of human‐Aβ load in the (D) hippocampus and (E) cortex using AI‐based HALO software, “Area Quantitation v2.4.3.” module. Data are represented as mean ± SEM, *N* = 4–5 per group of both sexes were analyzed for significance by one‐way ANOVA and Tukey's post hoc tests with *p < 0.05, **p < 0.01, ***p < 0.001, and ****p < 0.0001.

Additionally, the double KI of both *APP* and *PS1* familial AD mutations did not exhibit mutation‐induced artifacts, as observed by the stable expression of 22C11 (human and mouse Aβ antibody) reactive FL‐APP in both NOG and NAPS mice (Figure 1I,K). The lack of an antibody specific to human PS1 precluded the evaluation of the chimeric *PS1* gene expression. However, the physiological levels of *PS1* gene expression were confirmed by the lack of significant differences between levels of FL‐PS1 and PS1 CT fragments at 3, 6, and 9 months compared to age‐matched NOG control mice (Figure 1I,L,M). Additionally, the mutation‐induced differential APP cleavage was confirmed by the significantly increased levels of APP‐CTF‐β fragments in the NAPS mice (Figure 1I,N). APP‐CTF‐α remained comparable to NOG mice (Figure 2I,O).

### 3.3 Aβ pathology in single and double NA and NAPS KI mice

Amyloid pathology is the key driver of AD, which is recapitulated in the transgenic animal models. However, the random nature of gene insertion and the use of non‐endogenous gene promoters raise concerns about whether the observed amyloid phenotypes in these models result from familial AD mutation, overexpression, or both.^18^, ^41^, ^42^ To evaluate the development of amyloid pathology in the NA and NAPS mice, cortical tissue homogenates and 5 µm thick brain sagittal sections were analyzed for human Aβ development at 3, 6, and 9 months of age (Figure 2A). In cortical homogenates, both NA and NAPS mice show increased soluble and insoluble human Aβ_42_ at 3 months of age compared to NOG mice (Figure 2B). However, amyloid deposition was time‐limited to detect significant longer‐term age‐associated changes. Interestingly, *PS1* KI alone did not show the development of either soluble or insoluble human Aβ_42_. However, the addition of *PS1* mutation approximately doubled the amyloid load in NAPS mice compared to NA mice at all ages (Figure 2B,C). To further characterize the amyloid deposition in different brain regions, 5 µm sagittal brain sections stained for human‐specific Aβ antibodies (6E10) were quantitated. Compared to NOG mice, both NA and NAPS mice developed intraneuronal amyloid deposition as early as 3 months of age in both the cortex and hippocampus (Figure 2A,D,E). For an unbiased and reliable quantitation of the amyloid load using IHC, an AI‐based software HALO area quantitation module was utilized. Like the human Aβ_42_ ELISA (Figure 2B), the NAPS mice showed a consistent trend of significantly higher human amyloid deposition in the cortex and hippocampus compared to NA mice across all ages tested (Figure 2A,D,E). Although no significant hippocampal amyloid deposition was observed between NA and NAPS mice at 3 months, NAPS mice exhibited substantial increases in human amyloid load at 6 and 9 months of age compared to NA mice. At 9 months, NAPS mice showed more than a two‐fold increase in hippocampal amyloid load compared to age‐matched NA mice (Figure 2D). Significant cortical amyloid differences between NA and NAPS mice were observed only at 3 months of age. However, NAPS mice maintained a trend of higher amyloid load with age‐matched NA mice (Figure 2A,E).

In both NA and NAPS mice, we observed amyloid deposition primarily in the cortex and CA1, CA2, and CA3 hippocampus, with NAPS mice developing higher overall deposition compared to NA mice (Figure 2A). Interestingly, we did not observe amyloid deposition in the dentate gyrus region even in 20‐month NA or NAPS mice (data not shown). The amyloid deposits displayed a hierarchical progression, beginning in the cortex and expanding to the hippocampus. Cerebellar amyloid deposition was limited to Purkinje cells^43^, ^44^ (Figure S1). Vascular amyloid pathology, also known as cerebral amyloid angiopathy (CAA), was observed where amyloid deposits were in the walled cerebral blood vessels.

We evaluated brain sections for neurotoxic beta sheets and amyloid fibrils to confirm that the intraneuronal 6E10 anti‐amyloid antibody was not cross‐reactive CTF‐β or human APP. Thioflavin‐T is a fluorescent technique for beta‐sheet detections in amyloid deposits.^28^ Both NA and NAPS mice show thioflavin‐positive amyloid deposits, confirming the formation of neurotoxic beta‐sheet structures in the intraneuronal human Aβ deposits (Figure S2). Furthermore, brain sections were stained with anti‐amyloid OC fibril antibody that specifically binds to the oligomer‐specific conformation (OC) of the Aβ deposits and not to prefibrillar oligomers or natively folded proteins. Both NA and NAPS mice showed OC fibril‐positive staining, confirming the development of oligomeric human‐Aβ in both the NA and NAPS mice (Figure S2). These data confirm intraneuronal pathological amyloid deposits, with NAPS mice exhibiting significantly greater intraneuronal amyloid pathology than NA mice due to mutations. Mice developed only intraneuronal amyloid deposits and did not show reliable plaques, even in aged mice. However, antigen retrieval methods significantly influence the staining patterns and intensity of intraneuronal amyloid deposits and amyloid plaques. Studies have shown that intraneuronal amyloid reactivity is enhanced by heat but counteracted by formic acid antigen retrieval. However, formic acid retrieval develops significantly more intense amyloid labeling in plaques than in the heat method.^45^, ^46^ Interestingly, in NAPS mice older than 15 months, formic acid antigen retrieval led to the development of limited 6E10 reactive diffuse plaques (data not shown). However, no thioflavin reactivity was observed on the diffuse plaques. Consistent plaque pathology has not yet been observed and requires further characterization; the limited diffuse plaques detected were detected in NAPS mice older than 15 months. Additional APP FAD mutations will likely drive early extracellular plaque formation.

### 3.4 Microgliosis in single and double KI, NA, and NAPS mice

**FIGURE 3:**
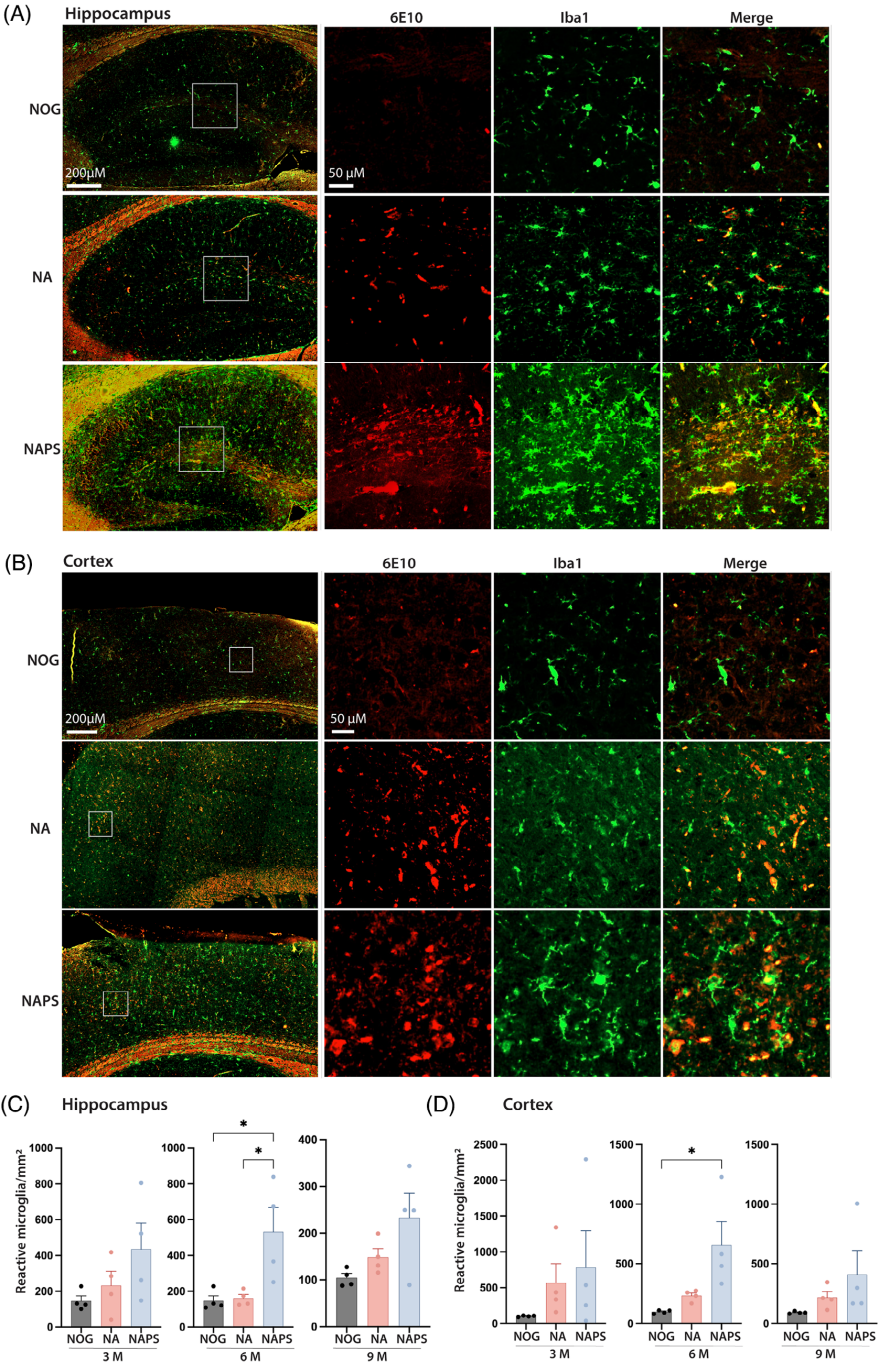
Characterization of microgliosis in single knock‐in (KI) APP^KM670,671NL^ (NA) and double KI APP^KM670,671NL^ x PS 1^M146V^ (NAPS) mice. Brains were harvested from 3‐, 6‐, and 9‐month‐old immunodeficient NOD/Shi‐scid/IL‐2Rγ^null^ (NOG), NA, and NAPS mice. Representative immunofluorescence (IF) images (5 µm sagittal sections) of microglia (Iba1, green) and human‐Aβ (6E10, red) from (A) hippocampus and (B) cortex of 6‐month‐old mice (scale bar = 200 µm). Areas of activated plaque‐associated microglia are highlighted by inserts showing human‐Aβ (6E10, red), microglia (Iba1, green), and merged images (red/green) (scale bar = 50 µm). Immunohistochemical (IHC) quantitation of activated microglia in (C) hippocampus and (D) cortex from 3‐, 6‐, and 9‐month‐old mice using the AI‐based HALO software, “Microglial Activation FL v1.0.6” module. Microglia with less than 15 processes and more than 15 µm^2^ of total process area were scored as activated. Data are represented as mean ± SEM, *N* = 4 mice per group of both sexes were analyzed for significance by one‐way ANOVA and Tukey's post hoc tests with *p < 0.05, **p < 0.01, and ***p < 0.001.

Human genetic and epidemiological data point out microglia and neuroinflammation's critical role in AD.^47^ To evaluate reactive microglia in the single (NA) and double (NAPS) KI mice, 5 µm sagittal brain sections were stained for Iba1 (green) and human amyloid 6E10 (red) (Figure 3A,B). For an unbiased and reliable quantitation of the reactive microglial phenotype, the Microglial Activation FL module of the AI‐based HALO software was utilized. Microglial cells with less than 15 processes and a total process area of more than 15 µm^2^ were considered activated. Considering the presence of intraneuronal amyloid deposition, both NA and NAPS mice demonstrated amyloid load‐dependent increases in reactive microglial in the cortex and hippocampus (Figure 3A,B). Consequently, there was a general trend of higher reactive microglia in NAPS mice compared to NA mice at 3, 6, and 9 months of age (Figure 3C,D). Notably, significant increases in reactive microglia were observed at 6 months in NAPS mice in both the cortex and hippocampus, highlighting a critical period of enhanced microglial response that needs further investigation. (Figure 3A,B).

### 3.5 Neuronal loss in single and double KI, NA, and NAPS mice

**FIGURE 4:**
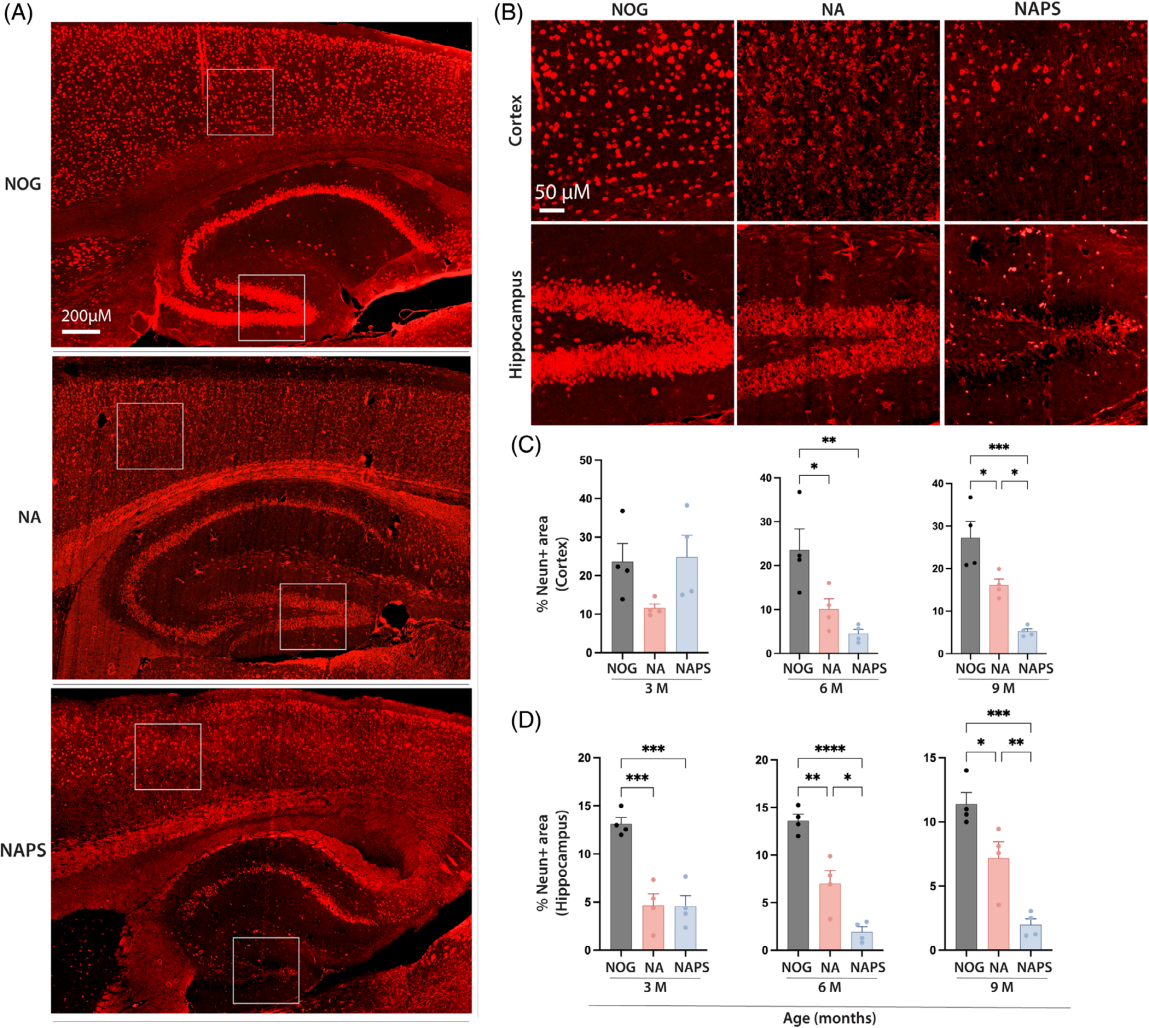
Characterization of neuronal loss in single knock‐in (KI) APP^KM670,671NL^ (NA) and double KI APP^KM670,671NL^ x PS 1^M146V^ (NAPS) mice. Brains were harvested from 3‐, 6‐, and 9‐month‐old immunodeficient NOD/Shi‐scid/IL‐2Rγ^null^ (NOG), NA, and NAPS mice. (A) Representative IF images (5 µm sagittal sections) of mature neuron (NeuN, red) staining of 6‐month‐old NOG, NA, and NAPS mice (scale bar = 200 µm). (B) Areas of neuronal loss in the cortex and hippocampus (dentate gyrus) are highlighted by inserts (scale bar = 50 µm). Immunohistochemical (IHC) quantitation of neuronal loss in the (C) cortex and (D) hippocampus using the AI‐based HALO software “Area Quantitation v2.4.3.” module. Data are represented as mean ± SEM, *N* = 4 mice per group of both sexes were analyzed for significance by one‐way ANOVA and Tukey's post hoc tests with *p < 0.05, **p < 0.01, and ***p < 0.001.

Brain atrophy caused by extensive neuronal loss is a prominent pathological feature of human Alzheimer's disease (AD) that is not well replicated in mouse models of the disease. Both NA and NAPS mice exhibit significant neuronal loss in the cortex and the hippocampus compared to NOG mice. The NeuN‐positive neuronal area in the cortex decreased by approximately 40% in NA mice and 70% in NAPS mice with advancing age (Figure 4A,B,C). Similarly, significant neuronal loss was observed across all hippocampus regions, with the most pronounced differences occurring in the hippocampal CA3 and dentate gyrus regions (Figure 4A,B,D). In older NAPS mice, a nearly complete depletion of neurons in the dentate gyrus was noted, which was more severe than the partial neuronal loss observed in NA mice.

**FIGURE 5:**
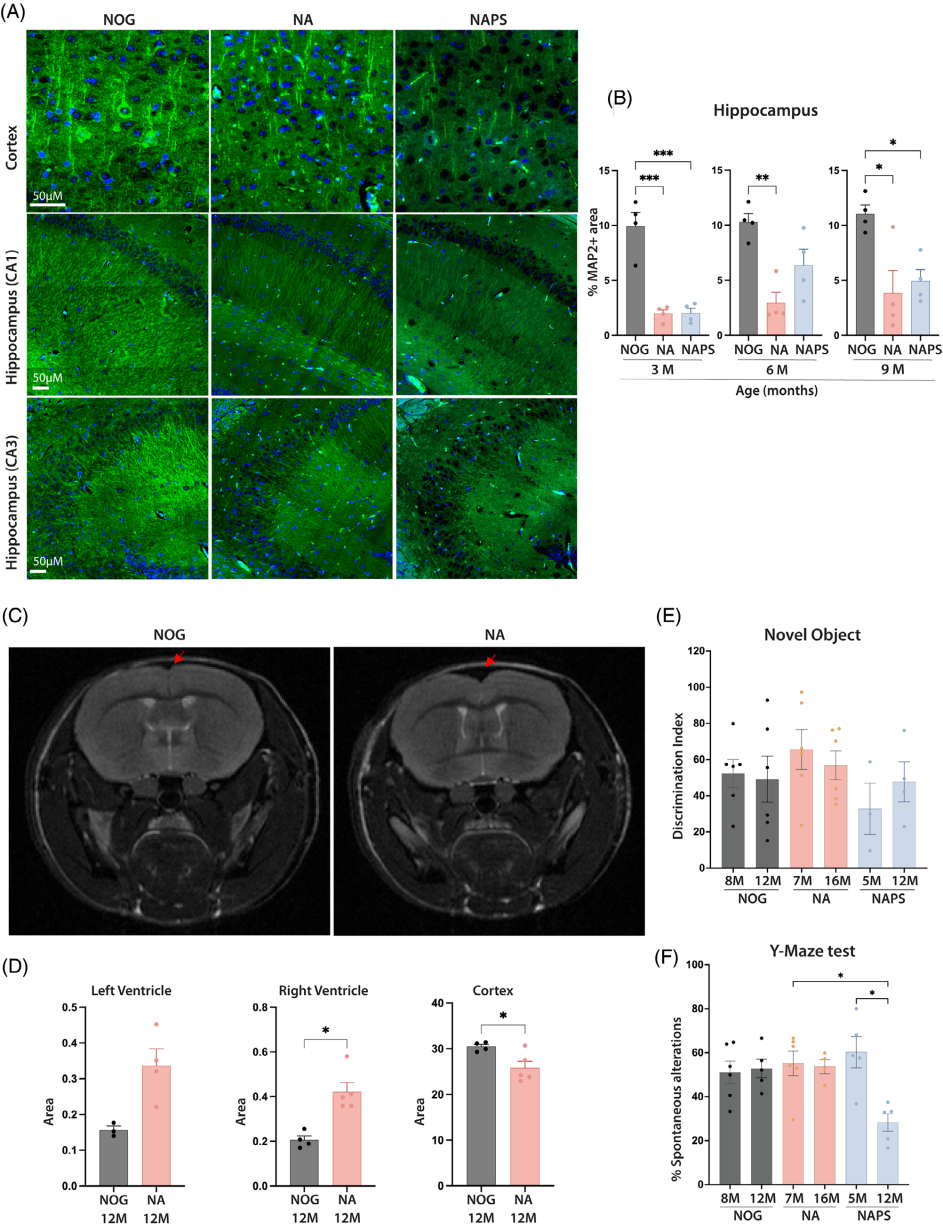
Characterization of neuronal integrity, brain atrophy and behavioral defects in single knock‐in (KI) APP^KM670,671NL^ (NA) and double KI APP^KM670,671NL^ x PS 1^M146V^ (NAPS) mice. Brains were harvested from 3‐, 6‐, and 9‐month‐old immunodeficient NOD/Shi‐scid/IL‐2Rγ^null^ (NOG), NA, and NAPS mice. (A) Representative immunofluorescence (IF) images (5 µm sagittal sections) of MAP2 (green) and nuclear (DAPI, blue) staining of 6‐month‐old NOG, NA, and NAPS mice (scale bar = 50 µm). (B) Immunohistochemical (IHC) quantitation of neuronal loss in the hippocampus using the AI‐based HALO software “Area Quantitation v2.4.3.” module. (C) Representative images of brain MRI scans showing loss of cortical volume (red arrow) in 12‐month‐old NA mice compared to age‐matched NOG control. (D) ImageJ quantitation of left and right ventricular area and cortical volume. (E) Quantifying discrimination index in the novel object test and (F) spontaneous alterations in the Y‐maze tests of NOG, NA, and NAPS mice at different ages. Data are represented as mean ± SEM, *N* = 4–6 mice/group of both sexes were analyzed for significance by one‐way ANOVA and Tukey's post hoc tests with *p < 0.05, **p < 0.01, and ***p < 0.001.

Brain sections were analyzed for loss of microtubule‐associated protein 2 (MAP2) in the hippocampus and cortex to corroborate the neuronal loss further and assess the extent of neurodegeneration. In the cortical region, both NA and NAPS mice demonstrated a decline in the MAP2‐positive area compared to age‐matched NOG controls, which were confined to the regions surrounding amyloid deposits (Figure 5A). Notably, the most significant loss in MAP2 density was observed in the hippocampus compared to age‐matched NOG mice (Figure 5A,B). Brain atrophy and reduced brain volume, resulting in notable neuronal loss, are key pathological features of AD. Brain MRI scans were conducted to evaluate brain volume loss. A substantial reduction in cortical volume was observed in NA mice at 12 months compared to age‐matched NOG mice (Figure 5C,D). Additionally, significant increases in ventricular volumes were identified in 12‐month NA mice, notably a marked increase in the right lateral ventricular volumes compared to those of NOG controls.

### 3.6 Cognitive defects in single and double KI, NA, and NAPS mice

Cognitive deficits are the hallmark of the clinical presentation of AD. We first evaluated visual recognition memory using the novel object recognition test. In this test, mice were presented with a familiar and a novel object and allowed to explore both freely. A control mouse is expected to spend more time with the novel object, thus exhibiting it by 12 months of age. These results suggest that a larger sample size and behavioral testing are necessary to detect more statistically significant differences.

### 3.7 PCA pathway and k‐clustering in KI, NA, and NAPS mice

**FIGURE 6:**
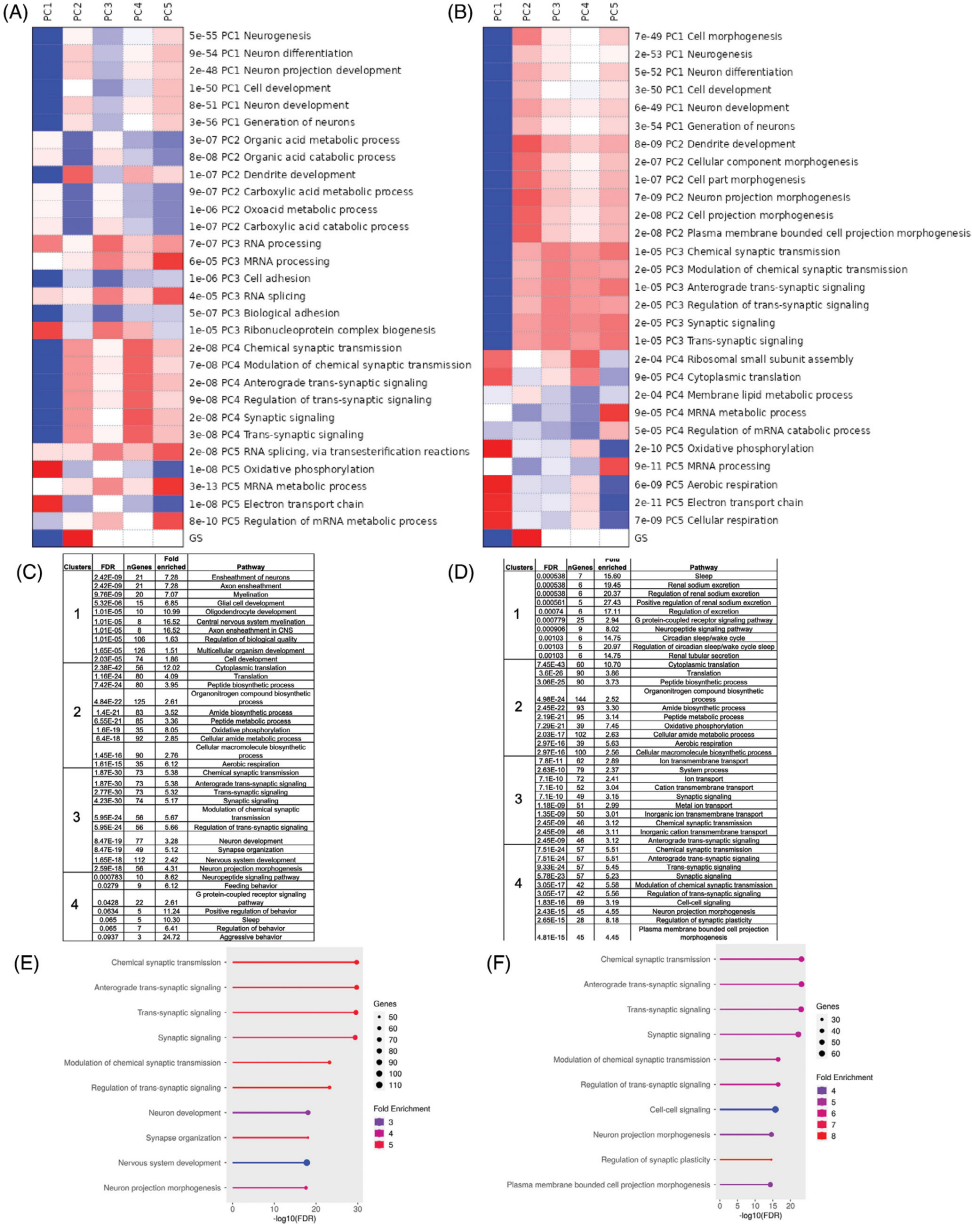
Principle component analysis (PCA) of the gene ontological changes in single knock‐in (KI) APP^KM670,671NL^ (NA) and double KI APP^KM670,671NL^ x PS 1^M146V^ (NAPS) mice. PCA pathway analysis of transcriptome from 3‐, 6‐, and 9‐month‐old (A) NA and (B) NAPS mice compared to immunodeficient NOD/Shi‐scid/IL‐2Rγ^null^ (NOG) mice. Pathways associated with k‐clustering in (C) NA and (D) NAPS mice. Lollipop plots demonstrating pathway enrichment in (E) clusters 1 and 3 for NA mice and (F) clusters 3 and 4 for NAPS mice. *p*‐Value ≤ 0.05 for *N* = 3–4 mice per group.

To investigate the differential gene expression from amyloid deposition, microgliosis, and neuronal loss, bulk RNA‐seq analysis was performed on transcripts extracted from cortical tissue in 3, 6, and 9 month‐old NA and NAPS mice and compared against that of age‐matched NOG mice. First, PCA (components 1–5) pathway analysis was performed to identify the primary source of variance in the bulk transcriptome from all age groups compared to NOG controls. The PC1 (the first principal component capturing the maximum variance) showed significant differences in the NA and NAPS mice captured by pathways involved in neurogenesis, neuron differentiation, neuron projection development, and generation of neurons (Figure 6A,B). In the PC2 and PC3 analysis, compared to NA mice, the NAPS showed further variance in pathways associated with synaptic dysfunction, axonal transport, and more severe neuroinflammatory responses (Figure 6A,B). The overall higher variance captured in the NAPS compared to NA mice underscores the compounded effect of the additional PS1 mutation. Next, k‐clustering of genes (top 2000) was performed, and four distinct clusters were identified in both NA and NAPS mice. Enrichment analysis of cellular component pathways (CCP) of each cluster showed that two clusters from both NA (Clusters 1 and 3) and NAPS mice (Clusters 3 and 4) had the highest number of genes and fold enrichment associated with neuronal function (Figure 6C,D). Pathway enrichment analysis of the clusters shows maximal changes in cellular component pathways, axon ensheathment in the central nervous system (CNS), synapse organization, and synaptic plasticity and signaling (fold change ∼ 5–8) (Figure 6E,F). Cluster 3 from NA mice and Cluster 4 from NAPS mice demonstrated maximal changes associated with neurons. Overall, NAPS mice show more significantly enriched pathways and fold enrichment than NA mice.

### 3.8 Enrichment pathways in NA and NAPS mice

**FIGURE 7:**
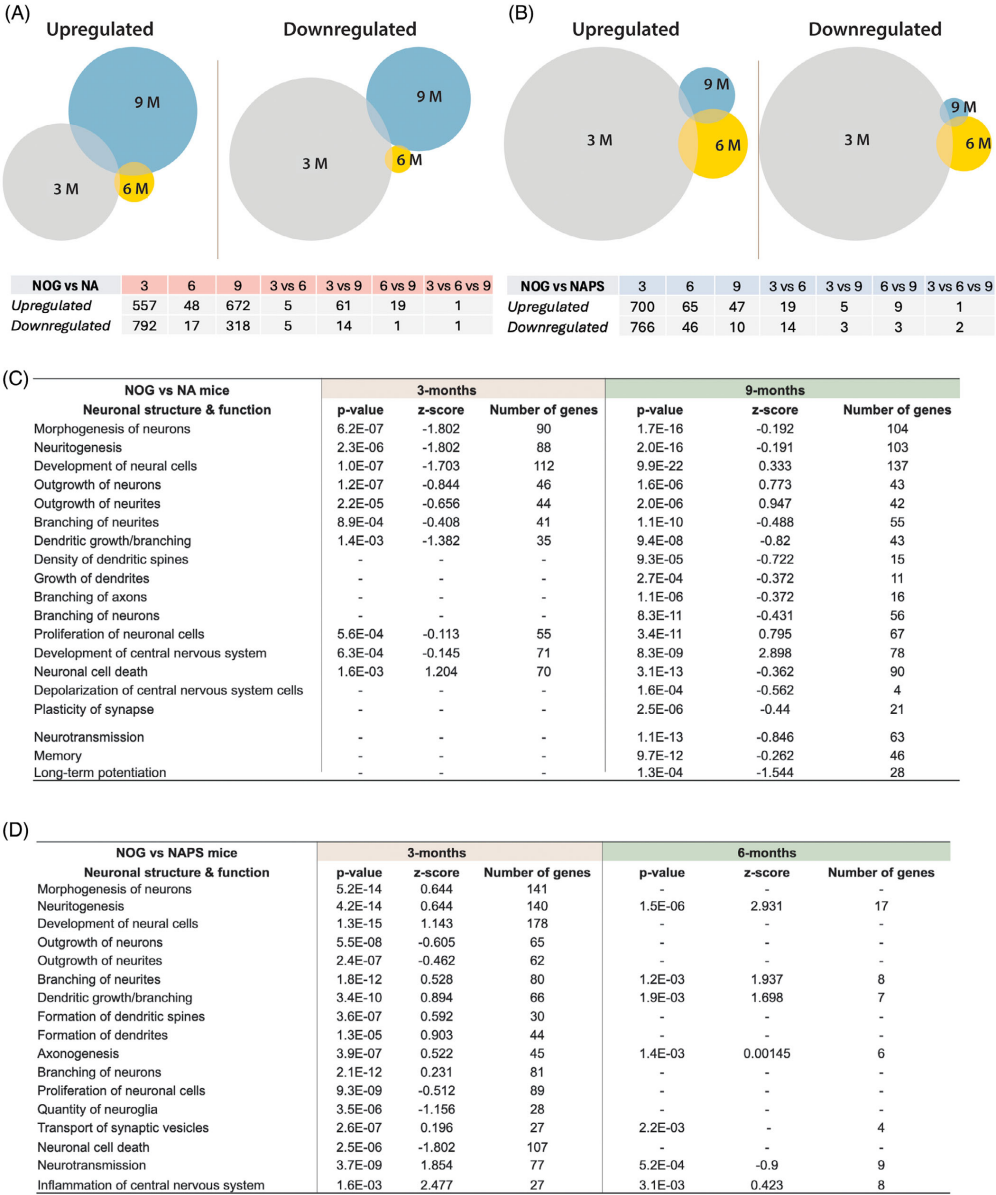
Enrichment pathway analysis in single knock‐in (KI) APP^KM670,671NL^ (NA) and double KI APP^KM670,671NL^ x PS 1^M146V^ (NAPS) mice. Venn diagram demonstrating significantly upregulated and downregulated genes in (A) NA and (B) NAPS mice at 3, 6, and 9 months compared to age‐matched immunodeficient NOD/Shi‐scid/IL‐2Rγ^null^ (NOG) mice. *p*‐Values, *z*‐scores, and number of genes represented in enrichment pathways associated with neuronal structure and function in (C) NA and (D) NAPS mice. *P*‐value ≤ 0.05 were considered significant for *N* = 3–4 mice per group.

Venn diagrams in Figure 7A,B illustrate the dynamic changes in gene expression at 3, 6, and 9 months of age in NA and NAPS mice compared to age‐matched NOG controls. In NOG versus NA mice, a significant (*p* < 0.05) number of genes were upregulated at 3 months (557), suggesting an early gene expression change in response to Aβ deposition. Interestingly, there was a more significant increase (673) in upregulated genes at 9 months, while comparatively fewer genes were upregulated at 6 months (48). A similar trend was observed in the downregulated genes, where significant gene changes were observed at 3 months (792) and 9 months (318) and comparatively fewer genes at 6 months (17) (Figure 7A). However, in the NAPS mice, significantly higher differential gene expression was observed at 3 months (700 up, 766 down), followed by fewer genes at 6 months (65 up, 46 down) and 9 months (47 up and 10 down) (Figure 7B). IPA canonical correspondance analysise (CCA) analysis demonstrated that the neuronal structural‐ and functional‐associated genes were mainly changed at 3 and 9 months of age in NA mice (Figure 7C). Results demonstrated downregulation of morphogenesis of neurons, the formation, extension, and branching of neurites (neuritogenesis), development of neural cells, outgrowth of neurites, dendritic growth/branching and proliferation of neuronal cells as demonstrated by negative *z*‐scores (activation scores) and *p*‐values (*p* < 0.05) in NA mice compared to NOG mice (Figure 7C). Interestingly, in addition to the pathways mentioned above, additional pathways—density of dendritic spines, branching of axons, the plasticity of the synapse, depolarization of the central nervous system, neurotransmission, memory, long‐term potentiation—were significantly (*p* < 0.05) downregulated, with negative *z*‐score in 9 months old NA mice. In the NAPS mice, maximal changes were observed at 3 months of age (Figure 7D). The CCA demonstrated downregulation of outgrowth of neurons and neurites, the proliferation of neuronal cells, the quantity of neuroglia, endocytosis of synaptic vesicles, and upregulation of neuroinflammation as demonstrated by z‐scores and *p*‐values (*p* < 0.05) in the NAPS group. Interestingly, it was shown that neurotransmission was significantly downregulated (*p* < 0.05, *z*‐score: −0.9) by 6 months in NAPS mice. However, all the genes associated with neurodegeneration and inflammation failed to demonstrate further changes over time. Details of the differentially expressed genes and the canonical pathways in KI NA and NAPS (Figures S3–S5).

### 3.9 Human immune reconstitution

**FIGURE 8:**
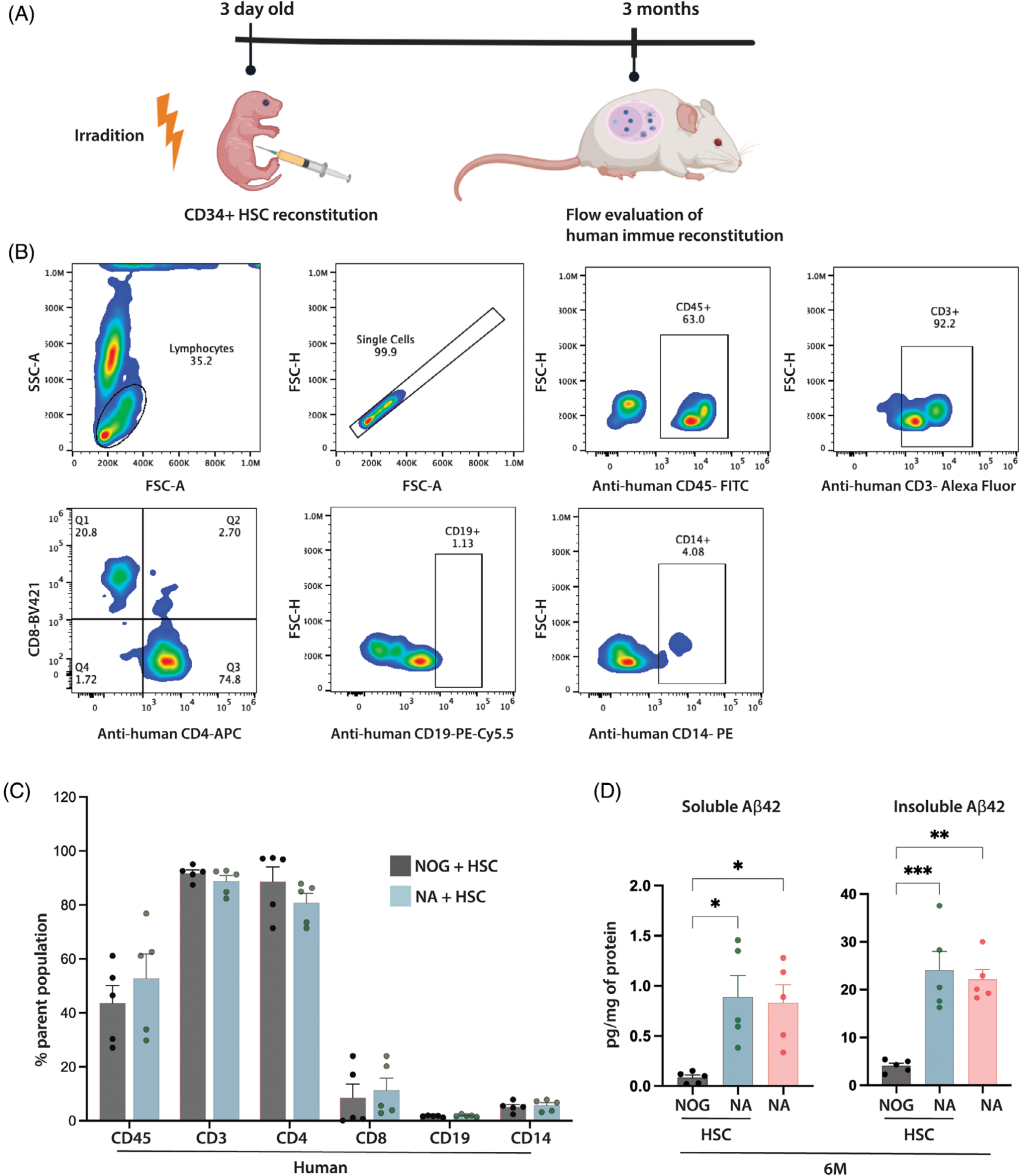
Human immune reconstitution of single knock‐in (KI) APP^KM670,671NL^ (NA) mice. (A) Schema representing the human immune reconstitution of NA mice. Representative (B) flow gating and (C) frequencies showing the development of human CD45+, and human T cells (CD3, CD4, CD8) and human B cells (CD19), and human macrophages (CD14) in NA mice. (D) Quantitation of human‐Aβ deposition by human Aβ_42_ ELISA using soluble (Tris‐HCl) and insoluble (Gu‐HCl) protein fractions of brain cortical tissue. Data are represented as mean ± SEM, *N* = 5 mice/group of both sexes were analyzed for significance by one‐way ANOVA and Tukey's post hoc tests with *p < 0.05, **p < 0.01, and ***p < 0.001.

To assess human immune reconstitution in KI mice, 3‐day‐old NA and NOG control mice were reconstituted with CD34+ HSC (Figure 8A). Flow cytometric evaluation of these mice at 3 months confirmed reconstitution of anti‐human CD45 positive immunocytes, including T cells (CD3, CD4, CD8), B cells (CD19), and macrophages (CD14). There were no significant differences between HSC‐reconstituted NOG and NA mice (Figure 8B,C). To investigate HSC reconstitution on the amyloid load, brain homogenates from 6‐month‐old HSC‐NA (human immune system reconstituted NA mice) and NA mice were analyzed by Aβ_42_ ELISA. Interestingly, HSC reconstitution did not alter the amyloid load compared to NA mice that were not reconstituted (Figure 8D). Long‐term studies are ongoing to further examine the effects of human immune reconstitution on amyloid pathology in both NA and NAPS mice.

## 4 DISCUSSION

Single (NA) and double (NAPS) KI mice were developed carrying the APP^KM670,671NL^ (Swedish) and PS 1^M146V^ human FAD mutations on immunodeficient NOG (NOD.Cg‐Prkdc^scid^ Il2rg^tm1Sug^/JicTac) mice. Sanger sequencing confirmed the targeted KIs of mouse *App* and *Ps1* homolog mutations, enabling endogenous promoter‐driven gene expression. The KI mice exhibited differential APP cleavage, resulting in CTF‐β expression. This was operative while maintaining physiological levels of full‐length APP. The pathobiological process led to intraneuronal human Aβ deposits with neurotoxic beta sheets and amyloid fibrils. These events resulted in amyloid‐associated microgliosis. Adding the *PS1* mutation in the NAPS mice doubled intraneuronal AD pathologies. However, no extracellular plaques were observed, even at 20 months of age. Despite this finding, the KI mice developed significant loss of NeuN+ neurons and dendritic architecture across the cortex and hippocampus. These processes led to brain atrophy in older mice—a unique feature compared to other KI and transgenic models, which develop neuronal loss around plaques.^48^ The KI mice can be reconstituted with a human immune system, offering a translationally relevant model for evaluating AD pathogenesis and immunotherapies. Timeline of AD pathology development is summarized in Figure **S6**.

The development of neurotoxic Aβ deposits and progressive neuronal loss are hallmarks of AD leading to memory deficits and brain atrophy in older patients.^49^, ^50^, ^51^ Recapitulating significant neuronal loss remains challenging in AD mouse models. Multiple transgenic and KI mice made available on immunocompetent backgrounds have demonstrated that more than one FAD mutation is required to induce early‐onset Aβ plaque pathologies. However, recent clinical evidence from AD and Down's syndrome patients suggests that intraneuronal Aβ could be an early event in AD pathogenesis.^52^ This role of intraneuronal Aβ formulation emerged after the development of 3xTg (transgenic for Swedish APP, P301L Tau, and M146 V KI mutation of PS1) and 5xFAD mice (transgenic for Swedish, Florida and London APP, and M146L and L286L PS1). These mice develop extensive intracellular Aβ before extracellular plaque is observed.^53^ The 3xTg mice develop intracellular Aβ up to 4 months and extracellular plaque by 12 months. These are recorded while additional mutations in 5xFAD mice accelerate intracellular Aβ at earlier times.^54^, ^55^ Interestingly, the prominent intraneuronal Aβ accumulation in 3xTg mice coincided with cognitive defects and synaptic abnormalities months prior to Aβ plaques.^53^ Additionally, both 3xTg and 5XFAD mice show reduced intracellular Aβ with advancing plaque pathology.^56^ 5xFAD mice develop intracellular Aβ fibrillation associated with neuronal death.^55^, ^57^ These animal models show distinct brain regions with either intraneuronal Aβ or extracellular plaques. Notably, regions with transient intraneuronal Aβ show neuronal loss compared to those with extracellular plaques.^58^, ^59^ These findings highlight an early neurotoxic phase of intraneuronal amyloid, which is shortened in mice with accelerated Aβ accumulation.

Herein, the Swedish APP KI mutation in an immunodeficient background (NA mice) leads to intraneuronal Aβ deposits, while adding the PS1^M146V^ mutation (NAPS mice) doubles the amyloid load. However, neither NA nor NAPS mice developed amyloid plaque, even at 20 months, retaining intraneuronal Aβ phenotype throughout their lifespan. These findings are consistent with earlier KI models, emphasizing the need for at least two APP mutations for plaque development. Substituting mouse Aβ with wild‐type human isoform (hAβ‐KI mice) produced OC+ Aβ granules but not amyloid plaques in the hippocampus, and adding PS1 ^M146V^ mutation increased the limited OC+ Aβ granules, but no plaques were observed.^60^ Single APP FAD mutation KI failed to develop plaque in APP^NL^ mice (Swedish) even at 24 months of age.^17^ However, KI of two APP FAD mutations led to plaques by 16 months in APP^NL‐F^ mice (Swedish and Iberian), and KI of three APP FAD mutations accelerated plaque formation to 6 months in APP^NL‐G‐F^ mice (Swedish, Arctic, and Iberian).^17^ While APP^NL‐G‐F^ mice developed the most robust amyloid plaques, neuroinflammation, and memory impairments in an age‐dependent manner, none of the KI mice were shown to develop broad neuronal loss.^17^

In contrast, NA and NAPS mice developed significant and progressive neuronal loss and loss of dendritic architecture across the cortex and hippocampus, driven solely by intraneural Aβ deposition. Characterization of these deposits confirmed the formation of neurotoxic beta sheets and Aβ OC+ fibrils. The extended intraneuronal Aβ phenotype in these models, contrasting sharply with transgenic mice like 3xTg mice where intraneuronal Aβ deposits are transient before progressing to amyloid plaques, likely leading to the observed broad neuronal loss. While spontaneous neuronal loss has been previously reported in NOG mice, the neuronal loss observed in these mice was significant compared to age‐matched NOG controls.^61^ However, we propose incorporating additional APP mutations (Arctic and Iberian), which have been shown to accelerate the transition from intraneuronal Aβ to plaques.^17^ Specifically, future studies aim to compare neuronal loss between NAPS mice carrying additional APP mutations—which are anticipated to have robust plaque formation and a shortened intraneuronal Aβ phase—and NAPS mice with only intraneuronal Aβ. This could determine if prolonged intraneuronal Aβ better models clinically relevant neuronal loss.

NA and NAPS mice lacked adaptive immune activities. Therefore, the unique pathology and absence of plaque development even in NAPS mice could be the result of an active innate immune system functioning in the absence of adaptive cellular responses. Interestingly, most transcriptomic change in these mice occurs early, at 3 months of age, with a marked decrease in differentially expressed genes by 9 months of age. This observation could point to the need for an adaptive immune system, which could either promote further development of pathology and/or protect neurons from pathological amyloid deposits.^62^ Indeed, our research and others have shown that Aβ‐specific effector T cells (Teffs) exacerbate AD pathology,^22^, ^63^, ^64^, ^65^ while Aβ‐specific Tregs attenuate disease progression in transgenic models.^21^, ^23^, ^66^, ^67^, ^68^, ^69^ The PCA of brain transcriptome across all ages showed that NAPS mice exhibited more pronounced changes compared to NA mice, highlighting the additional pathology from the PS1 mutation. Corroborating the broad neuronal loss observed in the KI mice, the key affected pathways include CNS development, neurogenesis, and synaptic signaling. Additionally, compared to NA mice, NAPS mice showed significant downregulation in neuronal structure and function and increased inflammation‐related genes by 3 months of age, which corroborates with the higher overall microgliosis and neuronal loss observed in these mice. While the bulk transcriptional shifts align with the AD pathology observed in the mice, further evaluation of the transcriptome changes via spatial transcriptomics or single‐cell sequencing will isolate the transcriptomic profile of the different AD‐relevant cell types and reveal potential biomarkers for neuronal loss and neuroinflammation. This study only describes the feasibility of human immune reconstitution of the NA mice; however, studies are underway to analyze the interplay between innate and adaptive immunity in AD pathobiology in human immune reconstituted mice.^70^

Furthermore, the development of amyloid‐associated behavioral defects is essential for AD preclinical models. The hippocampal intraneuronal deposits are noteworthy as they play a crucial role in memory consolidation and spatial navigation, comprising the cornu ammonis (CA1‐3) and dentate gyrus regions.^71^ Interestingly, NA and NAPS mice showed intraneuronal Aβ deposits in each of the hippocampal CA1‐3 regions. However, no deposition was observed in the dentate gyrus even in older mice. Although significant neuronal loss was observed in these mice, only NAPS mice developed limited behavioral defects at 12 months as determined by the Y‐maze test. The immunocompromised nature of the NOG mice presents unique challenges due to the altered physiological and immunological states. While more rigorous tests, such as Morris water maze, could potentially reveal behavioral defects, these studies were precluded by the susceptibility of the mice to infection and mortality. To accurately evaluate the behavioral defects in these mice, further investigation with larger cohorts of mice and more sensitive hippocampal behavior tests suitable for immunocompromised mice, such as contextual fear conditioning, Barnes maze, or electrophysiological tests that do not rely on baseline mouse behavior, are warranted.

Both NA and NAPS mice show novel early amyloid pathology, including neurotoxic intraneuronal Aβ and amyloid‐associated reactive microglia. Progressive and amyloid load‐dependent broad neuronal loss is a distinct feature of this model that is not replicated in other KI or transgenic mouse models and is only depicted in the KI rat model of AD.^16^ However, questions remain if the mice develop amyloid plaque with the KI of more APP mutations, spatial resolution of the Aβ deposition and neuronal loss, human disease similarity, behavioral defects, and pathology in HSC‐reconstituted mice. Additionally, the AD KI mice can be crossed with CD34‐NOG‐hIL‐34 mice (NOG mice with IL‐34 transgene that develops human‐like microglia in the brain after HSC reconstitution), which will allow the studies of human amyloid pathology in the context of the adaptive human immune system and human microglia.^27^ In summary, these mice allow, for the first time, evaluation of immunotherapies such as antigen‐specific human Tregs in the presence of a functional human immune system and human amyloid pathology. Additionally, the role of human infections in AD pathology can also be evaluated.^72^

## CONFLICT OF INTEREST STATEMENT

H.E.G. is a member of the scientific advisory board at Longevity Biotech and a co‐founder of Exavir Therapeutics, Inc. All other authors declare no conflicts of interest. Author disclosures are available in the supporting information.

## CONSENT STATEMENT

All human subjects provided informed consent. All authors read and approved the final manuscript.

## Supporting information



Supporting Information

Supporting Information

Supporting Information

## Data Availability

Data generated and analyzed from this study are included in this published article or its supplementary files.
