# Supplementary material for: Amyloid precursor protein and presenilin‐1 knock‐in immunodeficient mice exhibit intraneuronal Aβ pathology, microgliosis, and extensive neuronal loss
Source: Alzheimers Dement. 2025 Apr 7;21(4):e70084. doi: 10.1002/alz.70084 (PMC11975631; doi:10.1002/alz.70084)
Supplement: Supplementary file 2 — Supporting Information [file ALZ-21-e70084-s001.docx]

*Supplementary Materials*

## Amyloid precursor protein and Presenilin-1 knock-in immunodeficient mice exhibit intraneuronal Aβ pathology, microgliosis, and extensive neuronal loss

## Pravin Yeapuri^1*#^, Jatin Machhi^1*^, Emma G. Foster^1^, Rana Kadry^1^, Shaurav Bhattarai^1^, Yaman Lu^1^, Susmita Sil^1^, Roshan Sapkota^1^, Shefali Srivastava^1^, Mohit Kumar^1^, Tsuneya Ikezu^2^, Larisa Y. Poluektova^1^, Howard E. Gendelman^1#^, R. Lee Mosley^1^

*^1^Department of Pharmacology and Experimental Neuroscience, Center for Neurodegenerative Disorders, College of Medicine, University of Nebraska Medical Center, NE 68198, USA*

*^2^Molecular Neurotherapeutics Laboratory, Mayo Clinic, USA*

*Contributed equally

^#^**Corresponding Author:** Howard E. Gendelman (for review, revisions, and shared post-publication correspondences), Department of Pharmacology and Experimental Neuroscience, University of Nebraska Medical Center, Omaha, NE 68198-5800; phone 402-559-8920; fax 402-559-7495; email: [hegendel@unmc.edu](mailto:hegendel@unmc.edu)

Pravin Yeapuri (for shared post-publication correspondences), Department of Pharmacology and Experimental Neuroscience, University of Nebraska Medical Center, Omaha, NE  68198-5800; phone 402-559-4044; fax 402-559-7495; email: [pravin.yeapuri@unmc.edu](mailto:pravin.yeapuri@unmc.edu)


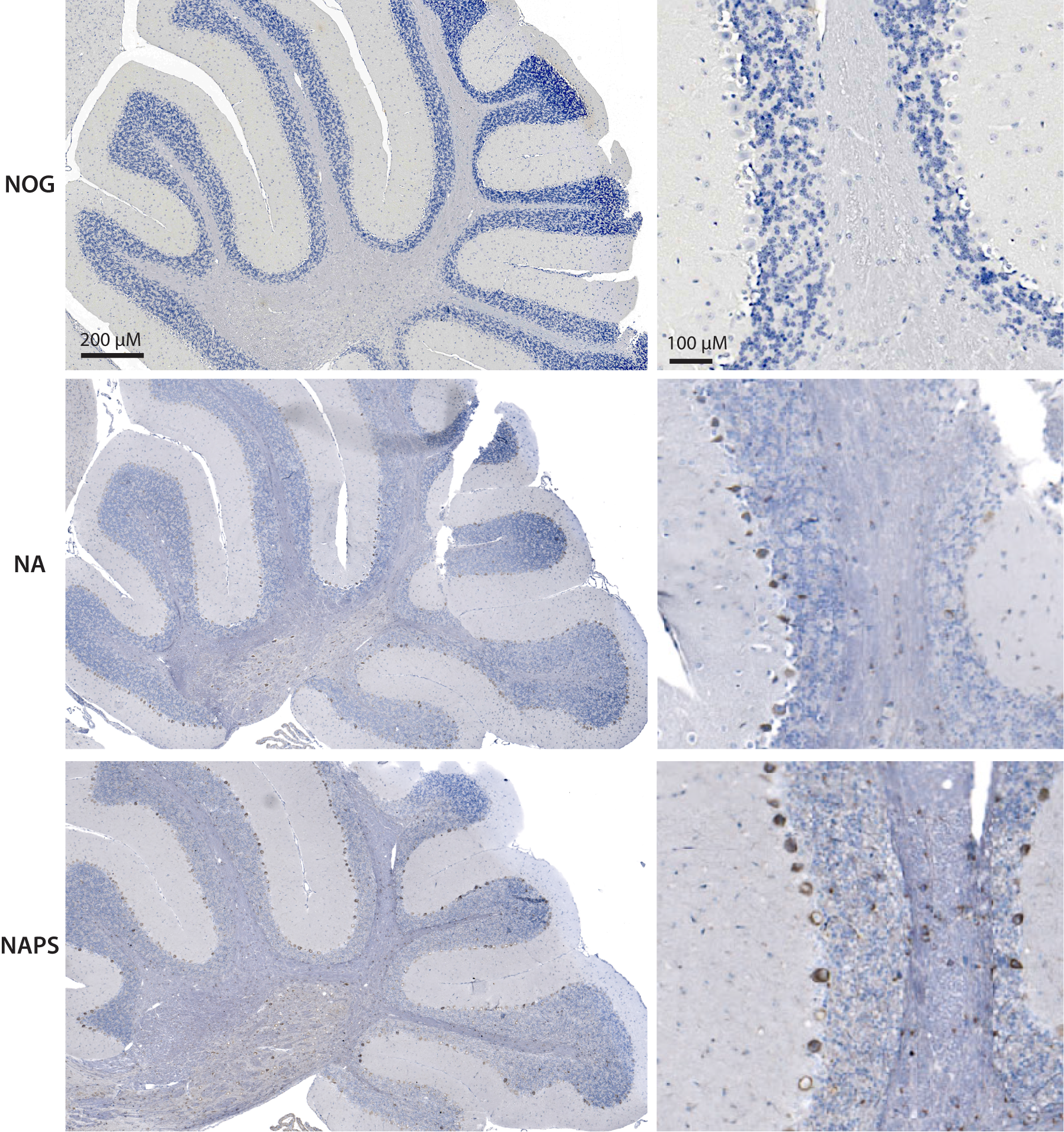


**Additional file 1: Figure S1: *Amyloid deposits in the cerebellum.*** Representative IHC images (5 µm sagittal sections) of human-Aβ deposition (6E10 antibody) in 6-month-old mice NOG, NA, and NAPS mice (Scale bar = 200 µM) using heat-induced epitope retrieval. Areas of Purkinje cells with 6E10 positive intracellular amyloid deposits are highlighted by inserts (scale bar = 100 µM).

*
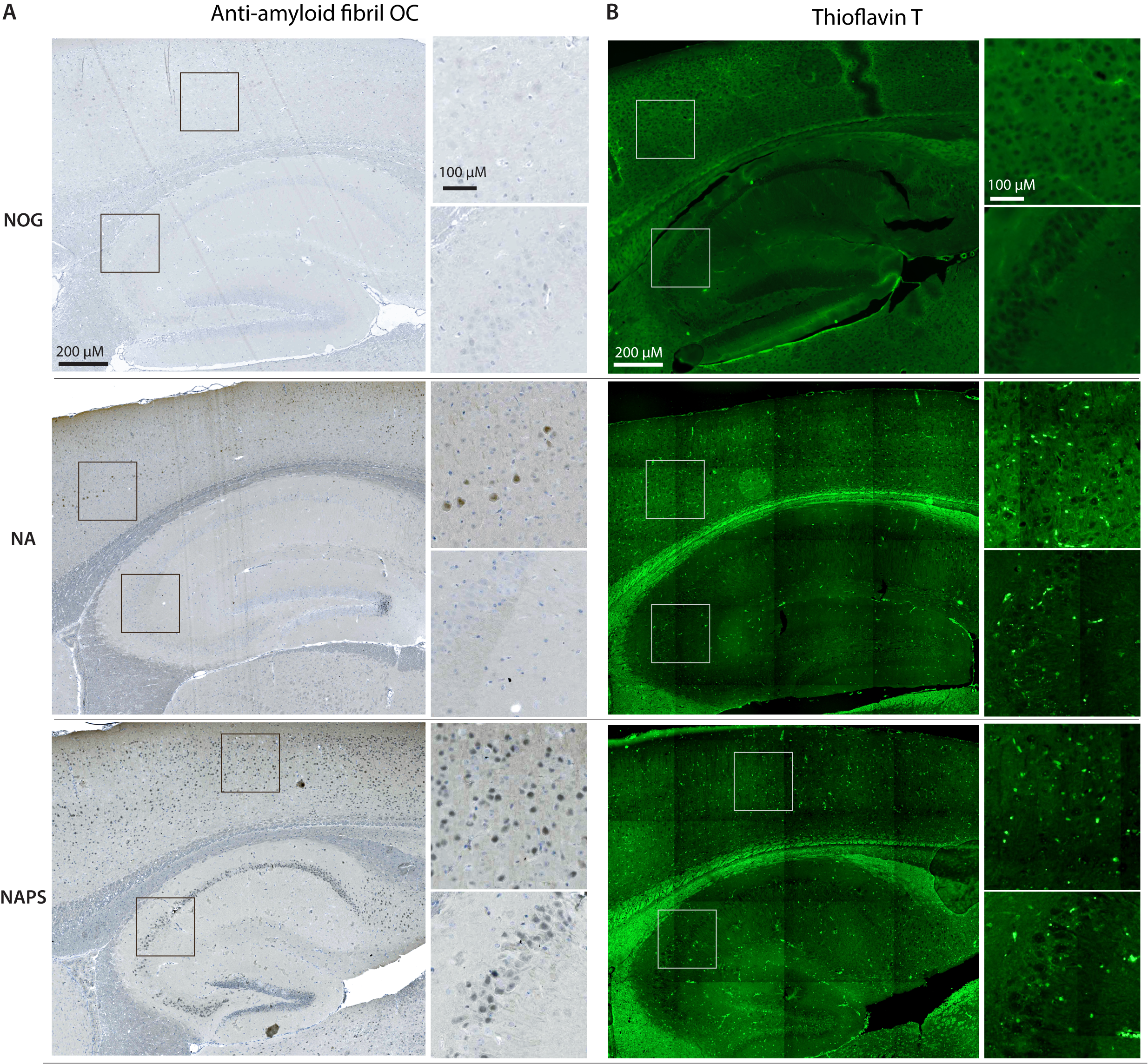
*

**Additional file 1: Figure S2: *Characterization of beta sheets in human-Aβ amyloid deposits in NA and NAPS mice.*** (**A**) IHC images (5 µm sagittal sections) of amyloid fibril OC antibody reactive human-Aβ deposition in 6-month-old mice (Scale bar = 200 µM) and deposition in the cortex and hippocampus are highlighted by inserts (scale bar = 100 µM) using heat-induced epitope retrieval (**B**) IHC images (5 µm sagittal sections) of Thioflavin T positive human-Aβ deposition in 6-month-old mice (Scale bar = 200 µM) and deposition in the cortex and hippocampus are highlighted by inserts (scale bar = 100 µM).

*
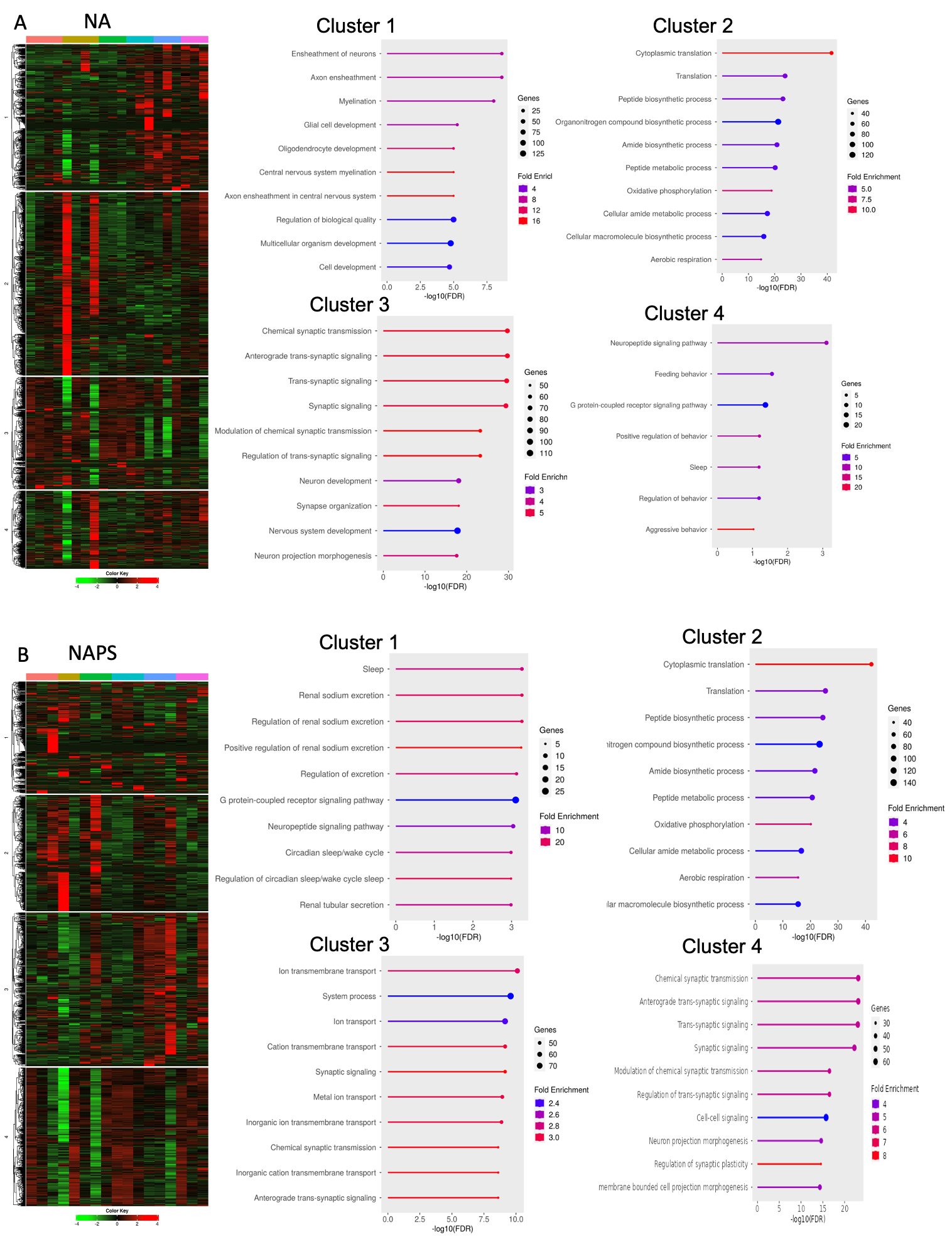
*

**Additional file 1: Figure S3: K-clustering and pathway enrichment analysis of gene expression in NA and NAPS mice:** Heat map displaying gene and enriched pathway clustering profiles of NA (**A**) and NAPS (**B**) mice. *P value ≤ 0.05 for N = 3-4 mice per group.*

*
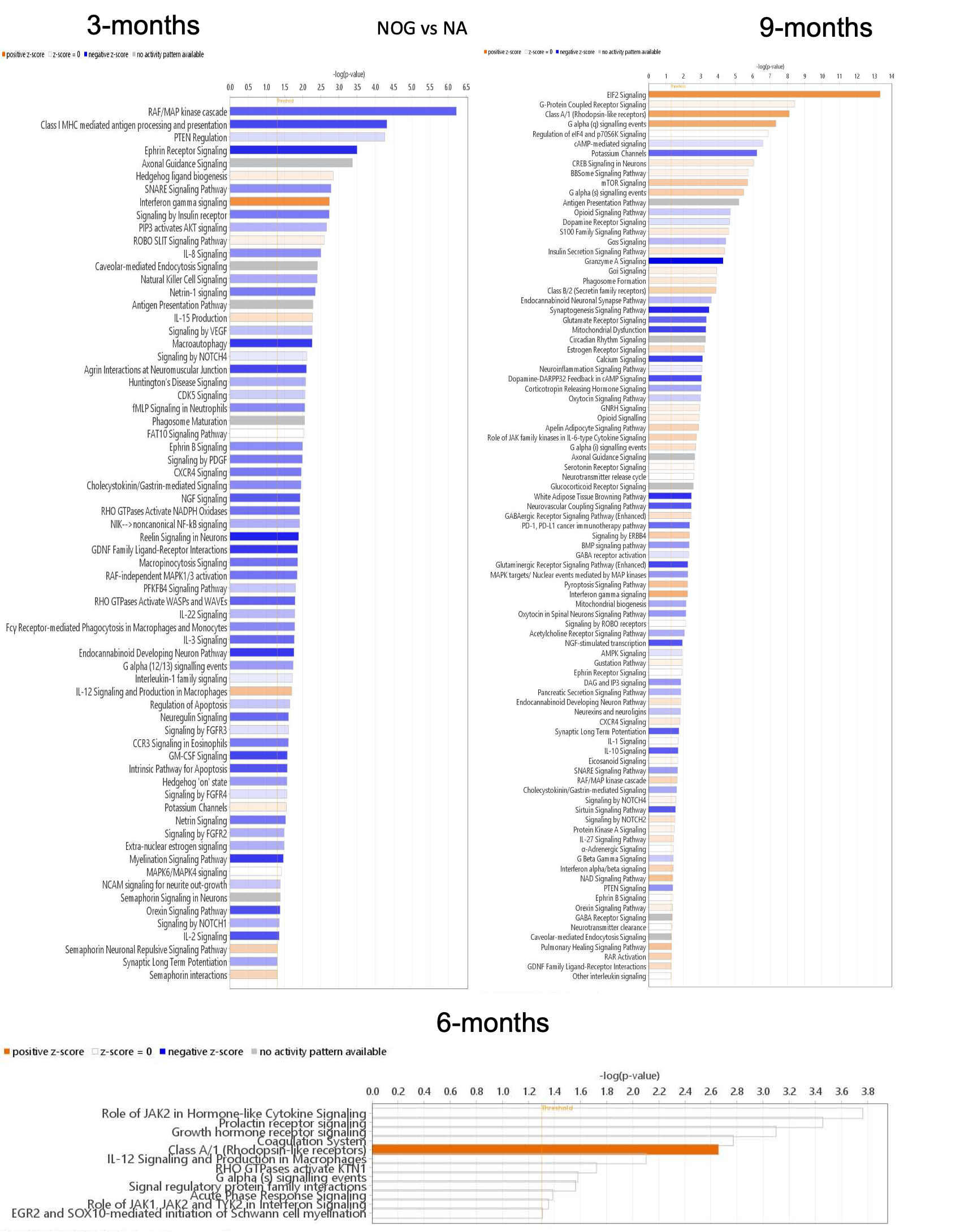
*

**Additional file 1: Figure S4: IPA Pathway analysis of gene expression in NA mice compared to age-matched NOG mice controls.** *P value ≤ 0.05 for N = 3-4 mice per group.*

*
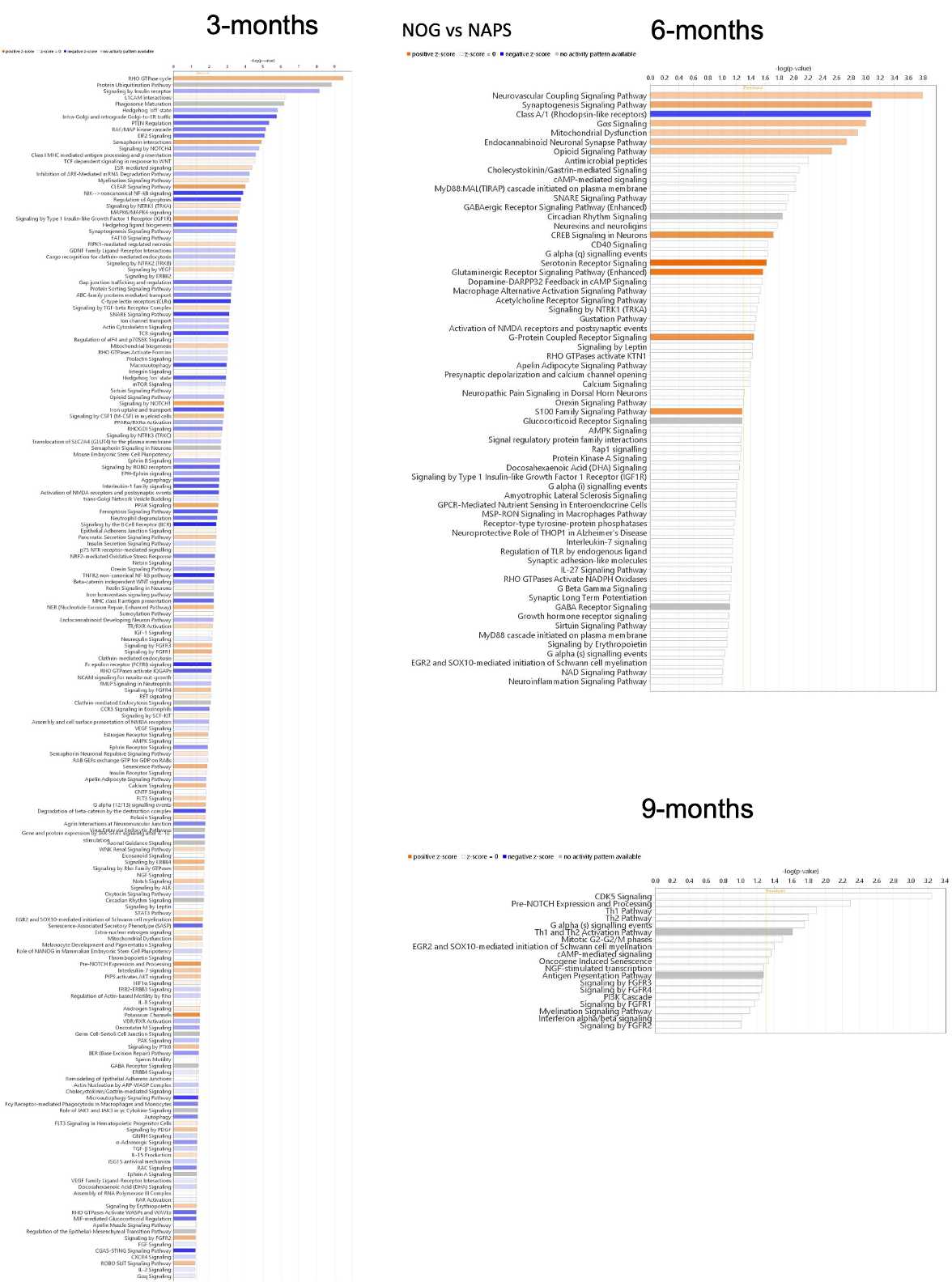
***Additional file 1: Figure S5: IPA Pathway analysis of gene expression in NAPS mice compared to age-matched NOG mice controls.** *P value ≤ 0.05 for N = 3-4 mice per group.*

**Additional file 1: Figure 6: Timeline of AD pathology development in NA and NAPS mice**
